# Exploring the mechanism of Cassiae semen in regulating lipid metabolism through network pharmacology and experimental validation

**DOI:** 10.1042/BSR20221375

**Published:** 2023-02-07

**Authors:** Lili Huang, Haiyan Zhu, Yuqin Tang, Zheng Luo, Luyun Xia, Chunjiang Zhang, Yanqiu Wang, Wenying Huai, Zhiyan Fang, Shenrong Li, Zhiyong Yan, Qiaozhi Yin, Tian-e Zhang

**Affiliations:** 1 School of Basic Medicine, Chengdu University of Traditional Chinese Medicine, Chengdu 611137, China; 2 School of Life Science and Engineering, Southwest Jiaotong University, Chengdu 611756, China

**Keywords:** Cassiae Semen, Free Fatty Acids, molecular docking, Network Pharmacology, non alcoholic fatty liver disease

## Abstract

Background: Multiple studies have assessed the role of Cassiae semen (CS) in regulating lipid metabolism. However, the mechanism of action of CS on non-alcoholic fatty liver disease (NAFLD) has seen rare scrutiny. Objective*:* The objective of this study was to explore the regulatory mechanism of CS on lipid metabolism in NAFLD. Methods*:* Components of CS ethanol extract (CSEE) were analyzed and identified using UPLC-Q-Orbirap HRMS. The candidate compounds of CS and its relative targets were extracted from the Traditional Chinese Medicine Systems Pharmacology, Swiss-Target-Prediction, and TargetNet web server. The Therapeutic Target Database, Genecards, Online Mendelian Inheritance in Man, and DisGeNET were searched for NAFLD targets. Binding affinity between potential core components and key targets was established employing molecular docking simulations. After that, free fatty acid (FFA)-induced HepG2 cells were used to further validate part of the network pharmacology results. Results*:* Six genes, including Caspase 3 (CASP3), phosphatidylinositol-4,5-bisphosphate 3-kinase, catalytic subunit α (PIK3CA), epidermal growth factor receptor (EGFR), and amyloid β (A4) precursor protein (APP) were identified as key targets. The mitogen-activated protein kinase (MAPK) signaling pathway was found to associate closely with CS’s effect on NAFLD. Per molecular docking findings, toralactone and quinizarin formed the most stable combinations with hub genes. About 0.1 (vs. FFA, *P*<0.01) and 0.2 (vs. FFA, *P*<0.05) mg/ml CSEE decreased lipid accumulation *in vitro* by reversing the up-regulation of CASP3, EGFR, and APP and the down-regulation of PIK3CA. Conclusion*:* CSEE can significantly reduce intracellular lipid accumulation by modulating the MAPK signaling pathway to decrease CASP3 and EGFR expression.

## Introduction

Cassiae semen (CS), the dried mature seed of the legume *Cassia obtusifolia* L. *or C. tora* L, was first documented in Shennong Bencao Jing. According to the 2020 edition of the Chinese Pharmacopoeia [1], CS protects the liver, brightens the eyes, and moisturizes the intestines. CS is one of the food and medicine homologous substances approved by the Ministry of Health in China [2]. As a type of tea, its value in protecting the liver and reducing body fat has been recognized [3]. CS attenuates lipid accumulation in white adipose tissues [4] and reduces the content of total cholesterol and triglycerides in the serum of hyperlipidemia models [5]. In modern clinical Chinese medicine, CS has been widely used to treat hyperlipidemia and diabetes [6–8]. One investigation observed no treatment-related adverse effects following sub-chronic toxicity evaluations of the ethanol extract of CS [9], suggestion that the extract is relatively safe. Meanwhile, Cassiae semen ethanol extract (CSEE) has been reported to have plasma lipid lowering effects in hyper-lipidemic rats [10] and improve pancreatic mitochondrial function in high-fat diet fed mice [11]. However, traditional Chinese medicine (TCM) and its combinations commonly have multiple targets, often resulting in diverse and complex action mechanisms. Therefore, further research on CS is required.

Lipid metabolism disorders may be the cause of many diseases, including non-alcoholic fatty liver disease (NAFLD), a clinicopathological syndrome diagnosed through macrovesicular fat accumulation in more than 5% of hepatocytes [12]. NAFLD is the hepatic manifestation of a metabolic syndrome and the leading cause of chronic liver disease in the world [13]. More than 25% of the worldwide population may be affected by NAFLD [14]. NAFLD has become the most prevalent liver disorder in the world but has yet to received sufficient attention. A significant number of patients remain undiagnosed and untreated because of the inadequacy of diagnostic tools and the absence of effective pharmacological therapies [15], and this has rendered the disease a major burden on the health system [16]. Fortunately, according to one report, all stages of NAFLD appear to be reversible with proper intervention [17]. However, until now, the U.S. Food and Drug Administration (FDA) has yet to approve any agent for the treatment of NAFLD. The discovery of traditional Chinese medicine-related drugs for NAFLD treatment has recently become the focus of research in this direction. It is, therefore, critical to explore the mechanism of action of CS in regulating lipid metabolism.

Network pharmacology, a frontier in the field of drug discovery, is valuable for unbiased investigations into the potential target space of natural products [18], and is, hence, consistent with the overall concept of traditional Chinese medicine and the multi-targeting approach of traditional Chinese medicine treatment. Network pharmacology provides a new method for studying the mechanism of TCM in the treatment of disease [19]. Combining experimental validation with network pharmacology could help accelerate TCM drug discovery and improve existing drug discovery strategies [20].

## Material and methods

### Analysis of the major components in Cassiae semen ethanol extract using UPLC-Q-Orbirap HRMS

Although water extraction is the traditional extraction method used for CS, recent research has shown [10,11] that CS extracted using ethanol has a more obvious effect on lipid regulation; therefore, ethanol was used as the medium to extract CS in this experiment. About 3 mg of CSEE was dissolved in 1.5 ml of 2 mg/ml acetonitrile solution, sonicated for 30 min, and then filtered with a 0.22 μm microporous membrane for component analysis. Liquid chromatography (LC) analysis was conducted on a Thermo Scientific Vanquish UPLC (Thermo Fisher Scientific, Massachusetts, U.S.A.), equipped with a Thermo Scientific Accucore™ C_18_ column (3 × 100 mm, 2.6 µm) at 30°C. The mobile phase consisted of 0.1% formic acid in water (A) and acetonitrile (B). The optimized gradient program was as follows: 0–5 min, 95–82% B; 5–10 min, 82% B; 10–15 min, 82–80% B; 15–16 min, 80–78% B; 16–18 min, 78–77% B; 18–27 min, 77–60% B; 27–32 min, 60–50% B; 32–36 min, 50–20% B; 36–40 min, 20–5% B. The flow rate was 0.3 ml/min, and the injected volume was 3 µl.

High-resolution mass spectrometry (HRMS) was performed using an electrospray ion source (ESI), and scanned in a positive ion mode employing a full scan/data dependent secondary scan (Full MS/dd-MS^2^). The parameters were optimized as follows: the capillary temperature and probe heater temperature were, respectively, 320 and 350°C in the negative and positive modes; the spray voltage was 3.5 kV in the positive mode, and 3.0 kV in the negative mode; sheath gas (N_2_) was 35 l/h, and auxiliary gas (N_2_, 99.9% purity) was 10 l/h; the max spray current was 100 A; the s-lens radio frequency level was 50.00%; and the full scan mode range was *m/z* 100–1500.

### Screening for active components and corresponding targets

The active components of CS were extracted from the Traditional Chinese Medicine Database and Analysis Platform (TCMSP) [21] (https://tcmsp.com/tcmsp.php). The thresholds for the filtering of the obtained active compounds were set as Oral Bioavailability (OB) ≥ 30 [22] and Drug-Likeness (DL) ≥ 0.18. In addition, the name of the compound in the TCMSP database was standardized in PubChem (https://pubchem.ncbi.nlm.nih.gov/) using InChlKey. Next, active compounds-related targets were screened with a standard ‘prob>0’ in the TargetNet (http://targetnet.scbdd.com/) [23] online databases and the top 15 targets [24] in the Swiss Target Prediction web (http://www.swisstargetprediction.ch/) server. The human gene symbol corresponding to the name of any protein target was transferred by using the Uniprot database (https://www.uniprot.org/) [25].

### Screening for NAFLD targets

The Therapeutic Target Database (TTD) (http://db.idrblab.net/ttd/) [26], Genecards (https://www.genecards.org), Online Mendelian Inheritance in Man (OMIM) (http://www.omim.org) [27], and DisGeNET (http://www.disgenet.org/) were used to screen for NAFLD-related targets. Targets with score >20 in Genecards [28] and score >0.8 in DisGeNET [29] were selected, after eliminating repeated targets, the protein targets associated with the disease were imported to the Uniprot data platform. The common targets of the compounds and the disease were displayed using the Vennny2.1 (https://bioinfogp.cnb.csic.es/tools/venny/) and were regarded as candidate genes.

### Construction of the drug-active compound-target-disease network

Cytoscape3.7.2 was used to construct and visualize the drug-active compound-target-disease network. The nodes in the network diagram, represent different targets and components. Cytoscape was subsequently employed to calculate the average shortest path length, betweenness centrality, closeness centrality, neighborhood connectivity, and the degree between each node using its algorithm. The calculated degree is same as the number of directed edges.

### Construction of the protein–protein interaction network

Candidate genes were entered into the Retrieval of Interacting Genes online analysis [30] software (STRING, https://www.string-db.org/) to construct the protein–protein interaction network (PPI). In the STRING database, the minimum required interaction score necessary to screen with medium confidence (0.4) in the human species and the active interaction sources was dependent on texting, experiments, database, co-expression, neighborhood, gene fusion, and co-occurrence. The visual network diagram was created using the Cytoscape software [31].

### Enrichment analysis of the KEGG pathways

The Kyoto Encyclopedia of Genes and Genomes (KEGG) pathway enrichment analysis of candidate genes was performed using the Cytoscape software plugin tool Clue GO [32], where a *P*-value ≤ 0.05 was considered to indicate a biological signal pathway with a unified scientific meaning. In the analytical process, the nodes represent terms that are linked based on a predefined kappa score level. The initial kappa score level threshold can be adjusted from 0 to 1 to restrict network connectivity in a customized way [33]. And the terms with at least three genes can be displayed.

### Molecular docking verification

The energy required for core compounds to bind with key targets was evaluated using molecular docking simulations. The top 6 targets in the PPI network according to the degree value and the top 7 components of CS were selected for molecular docking. After downloading the MOL2 files of the seven main active ingredients from the TCMSP and entering the target into the Protein Data Bank (PDB) (http://www.rcsb.org/), the best protein structure was selected and saved in pdb format. The gained protein structure and active ingredients were then imported into PYMOL for pretreatment such as with water or ligand removal. After pretreatment, they were docked using the AutoDuck Vina software. The main connection mode between small molecules and large proteins was established by introducing the PDB file into LIGPLOT software.

### Preparation of CSEE and FFA

CS was purchased from Chengdu university of TCM in Sichuan, China (Lot No.200700651), and was identified by Professor Jinlin Guo, School of Pharmacy, Chengdu University of TCM. The powder of Cassiae semens (10 g) was extracted using 95% ethanol at 90°C for 3 h under a reflux condensation device. The acquired product was filtered, and the filtrate was concentrated for 40 min and lyophilized using a freeze-drying system [9]. After weighing the collected dry residue at 0.812 g (yield: 8.12%), the dry residue was dissolved in dimethyl sulfoxide (DMSO) to a final concentration of 500 mg/ml, stored at −40°C until use after aliquoting into several tubes. Free fatty acid (FFA) was prepared by mixing oleic acid with palmitic acid at a ratio of 2:1.

### Cell culture and treatment

HepG2 cells were cultured in 90% high sugar Dulbecco’s Modified Eagle Medium (Gibco, Thermo Fisher Scientific, Beijing, China), 10% fetal bovine serum (Every green, Tian Hang Biotechnology Co, Zhejiang, China), and 1% antibiotic (streptomycin 100 U/ml and penicillin 100 U/ml (Biosharp, Labgic Technology Co, Beijing, China), and maintained at 37°C under 5% CO_2._

### Cell viability in HepG2 cells

HepG2 cell viability was assessed using a Cell Counting Kit-8 (Biosharp, Labgic Technology Co, Beijing, China). HepG2 cells (2 × 10^3^ per well) at logarithmic phase were seeded in 96-well plates at 100 µl per well and treated with different concentrations of CSEE and/or FFA for 24 h. After the treatment procedure, the liquid in the wells was removed, and 100 µl of complete medium mixed with 10 µl of CCK-8 solution was introduced into each well. The mixture in the plate was incubated at 37°C for 2 h [34], shaken, and the absorbance was measured at 450 nm using a microplate reader.

### Oil Red ‘O’ (ORO) staining

HepG2 cells were washed with phosphate-buffered saline (PBS), fixed in 4% paraformaldehyde for 30 min, stained with the ORO working solution for 30 min at 37°C, and then rinsed three times with PBS. The cells were then subjected to the qualitative analysis of lipid using a microscope. The quantification analysis of lipid accumulation was performed based on OD values. A 0.5% storage solution of ORO was prepared by mixing 0.25 g of dry oil red power with 50 ml of isopropanol and then filtering the mixture using a 0.22 µm membrane. A working solution of ORO was concocted by blending the storage solution with deionized water at a ratio of 3:2.

### Quantitative real-time- PCR

The total ribonucleic acid (RNA) from each group of HepG2 cells was extracted utilizing the Total RNA Extraction Reagent (Mei5bio, Beijing, China), and cDNA was synthesized using HiScript II RT SuperMix for qPCR (Vazyme, Nanjing, China). The SYBR green qPCR Master Mix (Vazyme, Nanjing, China) was employed for the quantification of the gene expression levels. The succinate dehydrogenase complex flavoprotein subunit A (SDHA) acted as an internal reference, and the 2^−ΔΔCT^ method was used to determine relative fold changes in gene expression. Designed primers sequences are shown presented in Supplementary Table S1.

### Statistical analysis

Data were analyzed using GraphPad Prism 8.3.0 (GraphPad Software, San Diego, CA, U.S.A.). One-way ANOVA with Tukey’s multiple comparisons test was used to compare the effects of different concentrations of CSEE on cells in the *in vitro* experiments. There were only independent single variables (concentration) in the whole experiment. All data were presented as the mean ± S.D. *P*<0.05 was considered statistically significant.

## Result

### UPLC-Q-Orbirap HRMS analysis of Cassiae semen ethanol extract

**Figure 1:**
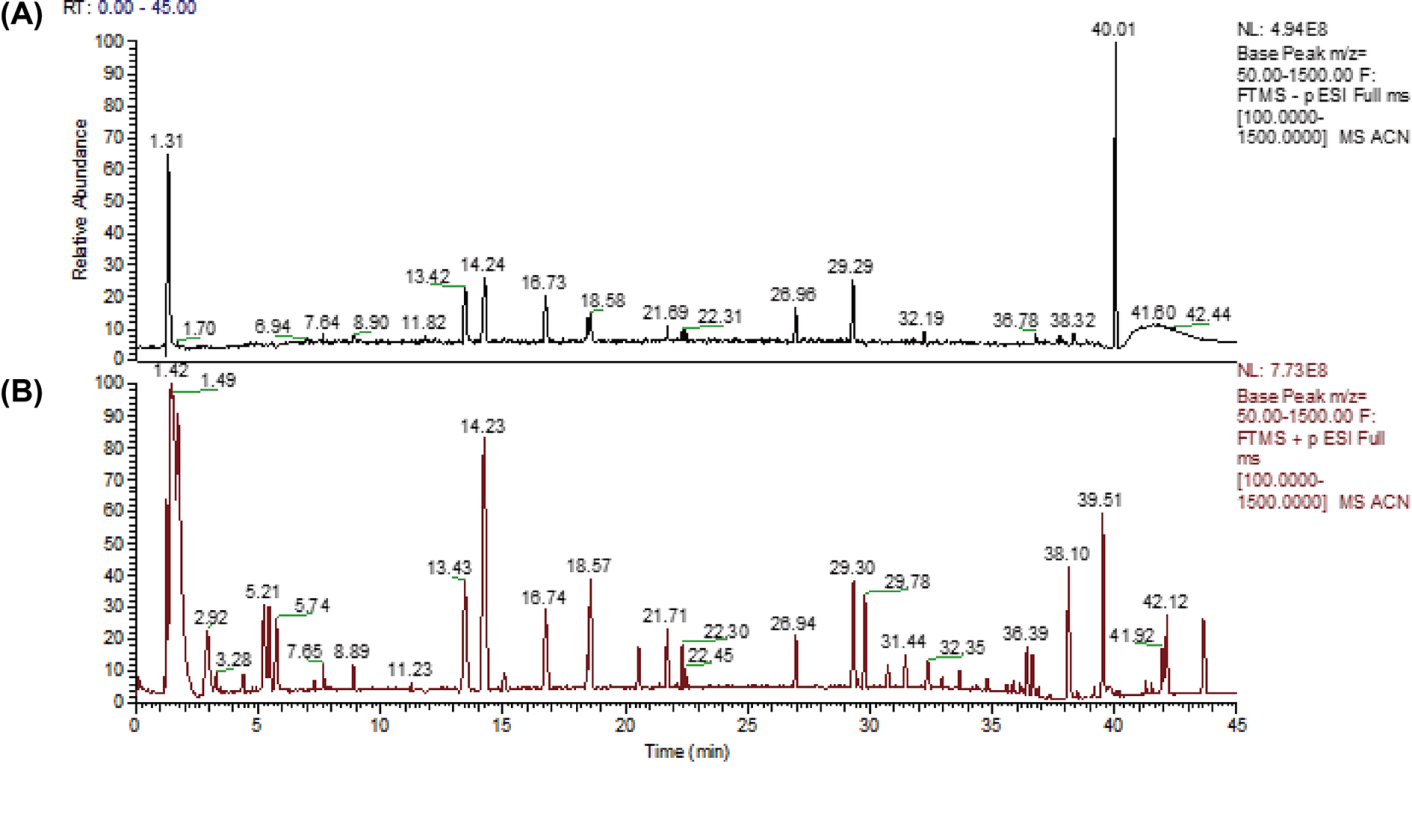
The main component in CSEE was detected by UPLC-Q-Orbirap HRMS (**A**) Negative ion mode total ion chromatogram (TIC). (**B**) Positive ion mode TIC.

The chemical constituents in traditional Chinese medicine are crucial for finding active ingredients and determining their material bases. In the present study, a full scan was performed in positive and negative ion modes using the UPLC-Q-Orbirap HRMS platform. Findings from the scan showed that, CSEE had abundant chromatographic peaks and good chromatographic curves in positive ion mode, with most of the compound presenting [M + H] ^+^ ions. (Figure 1).

The collected raw data were imported into the Xcalibur™ software for peak alignment and peak extraction operations. The possible molecular formulas were then fitted with the extracted molecular ion chromatographic peaks and isotope peaks and combined with the mzCloud network using the measured spectra of secondary fragments. The filtering parameters were set as follows: the peak area threshold was 80,000, the primary and secondary mass deviations were 5 ppm, and the matching score was higher than 80. This analysis identified a total of 56 components, including toralactone and quinizarin and other components that we previously screened. Mass spectrometric data on the proposed name of the identified compounds, the calculated retention times, molecular formula, molecular weight, and MS^2^ fragmentation patterns are presented in Supplementary Table S2.

### Results of active compounds and related targets gene

While 68 compounds of CS were obtained from the TCMSP database, only 14 main active ingredients were acquired based on OB ≥ 30% and DL ≥ 0.18. The Mol ID, molecule name, structure, and corresponding OB and DL values of the information of the active compounds are shown in Table 1. Importing the protein targets corresponding to the active ingredients into the Uniport data platform yielded 313 target genes of active compounds; duplicates were removed (details are in Supplementary Table S3). Additionally, 1429 NAFLD-related targets were extracted from TTD, Genecards, OMIM, and DisGeNET databases after deleting duplicates (details are provided in Supplementary Table S4). In the end, 64 candidate genes of CS for the treatment of NAFLD were illustrated on a Venn diagram (details are Supplementary Table S5).

**Table 1:** Active compounds of Cassia semen and their parameters

Mol ID	Molecule name	Structure	OB (%)	DL (%)
MOL002268	Rhein	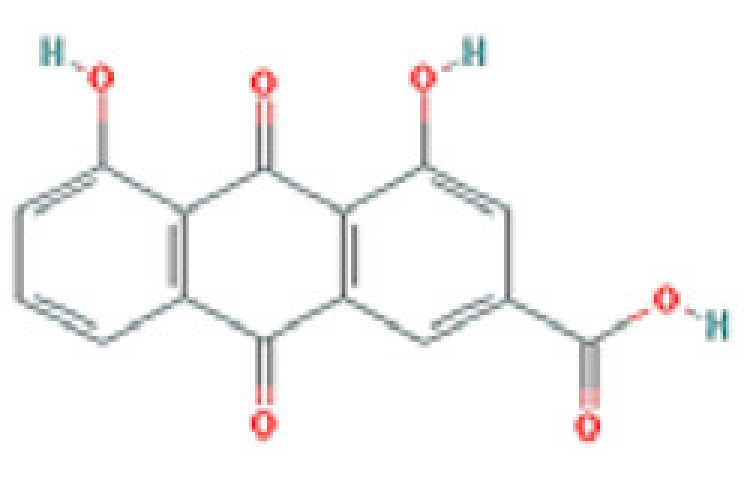	47.07	0.28
MOL006481	Gluco-obtusifolin	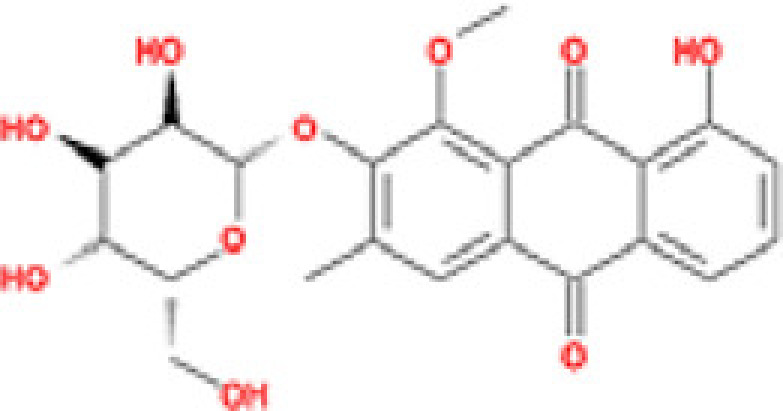	42.41	0.81
MOL006466	Rubrofusarin	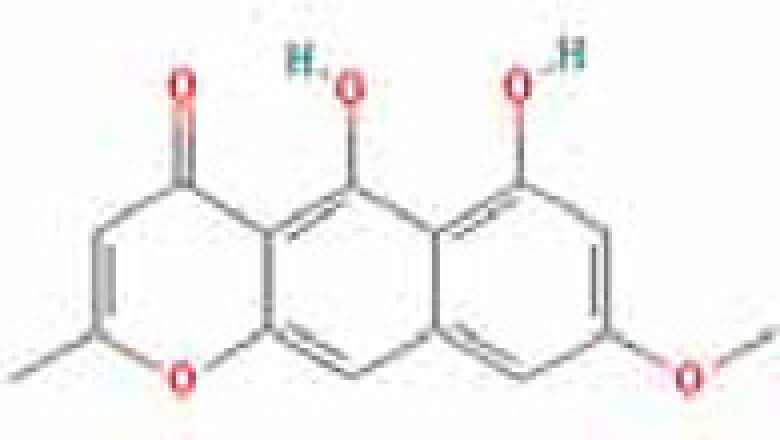	45.55	0.24
MOL006475	Obtusin	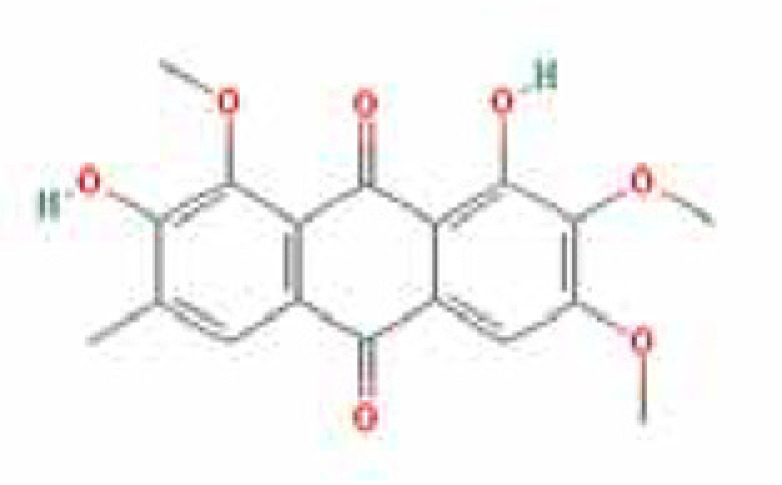	81.43	0.4
MOL005043	Campesterol	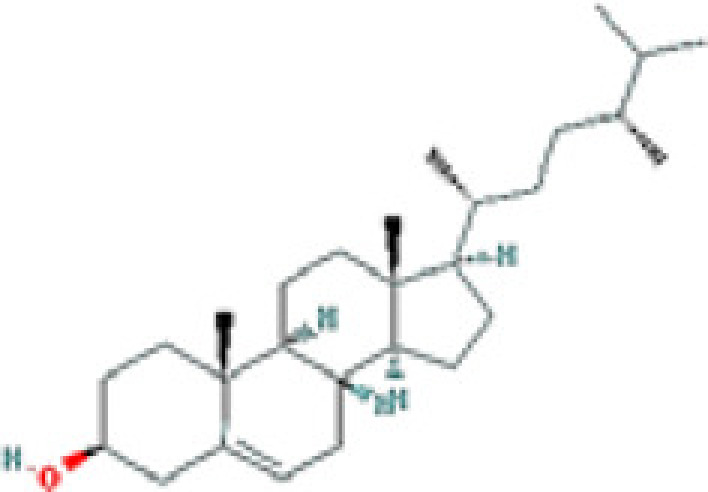	37.58	0.71
MOL000471	Aloe-emodin	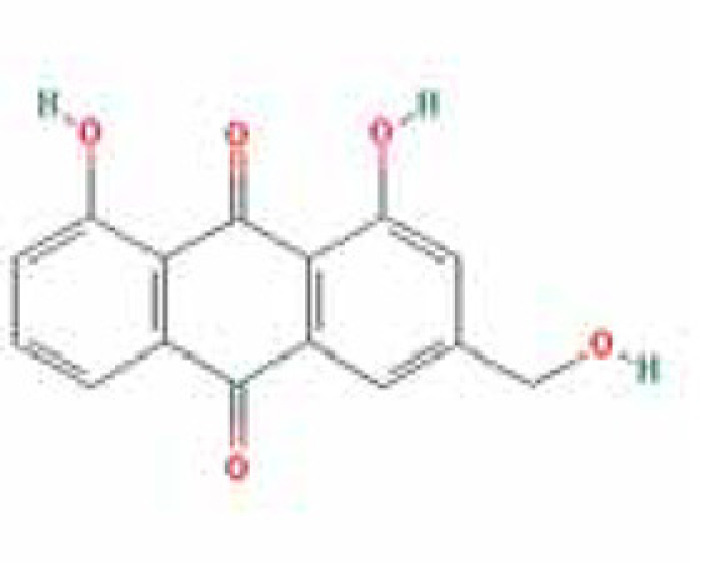	83.38	0.24
MOL002281	Toralactone	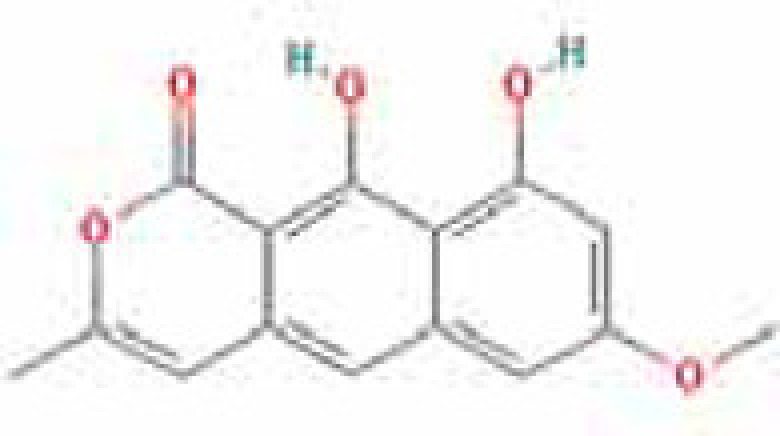	46.46	0.24
MOL000449	Stigmasterol	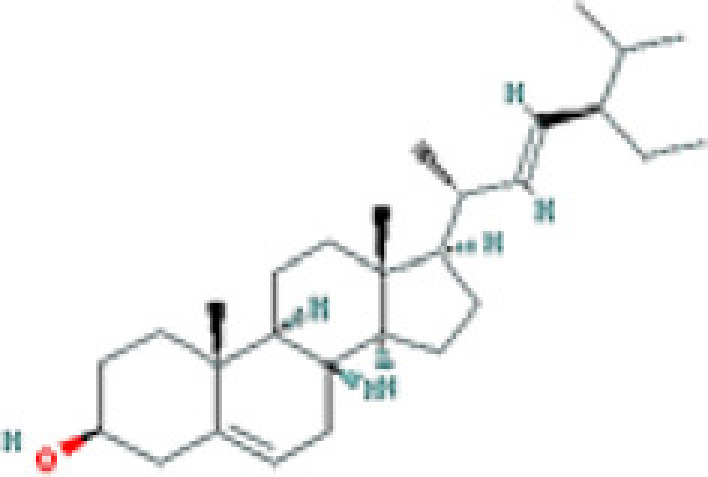	43.83	0.76
MOL006472	Aurantio-obtusin	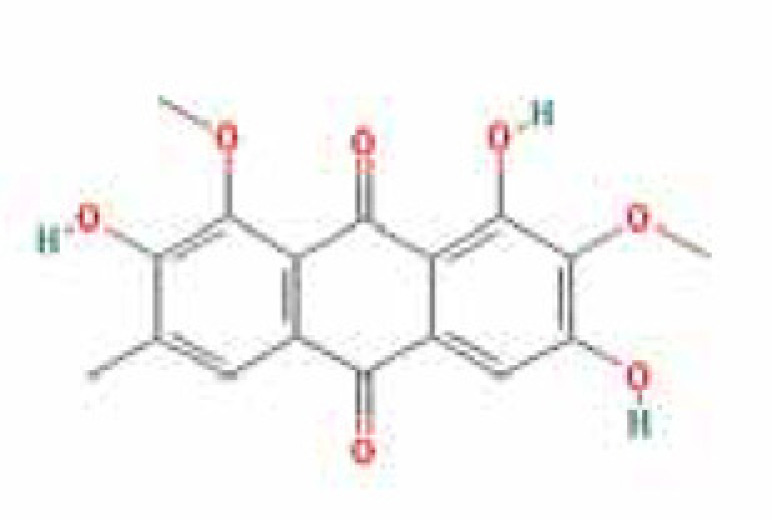	31.55	0.37
MOL006482	1H-Naphtho(2,3-c) pyran-1-one, 3,4-dihydro-9,10-dihydroxy-7-methoxy-3-methylene-	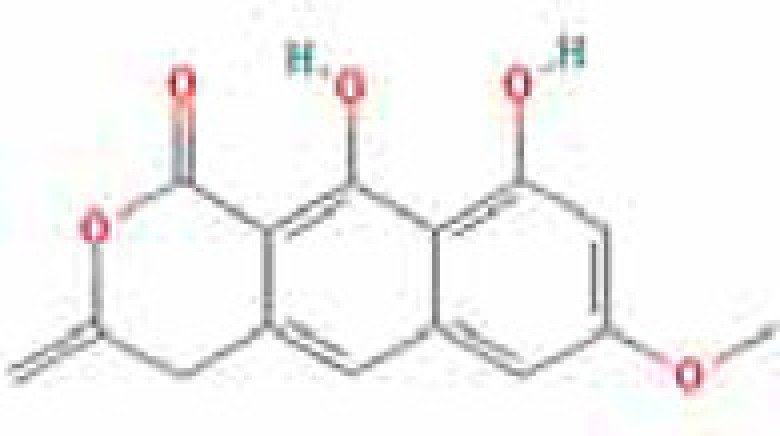	63.25	0.24
MOL000953	Cholesterol	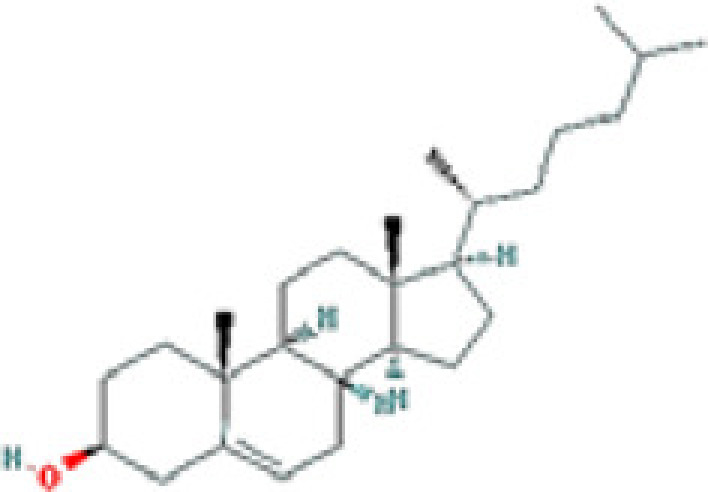	37.87	0.68
MOL006486	Obtusin	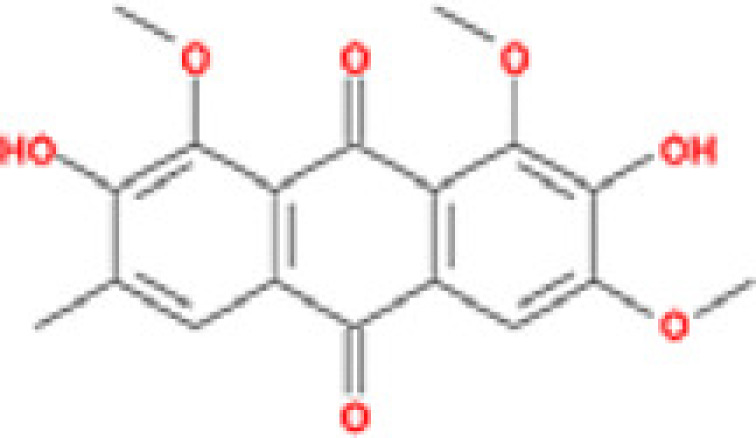	31.24	0.4
MOL006489	Quinizarin	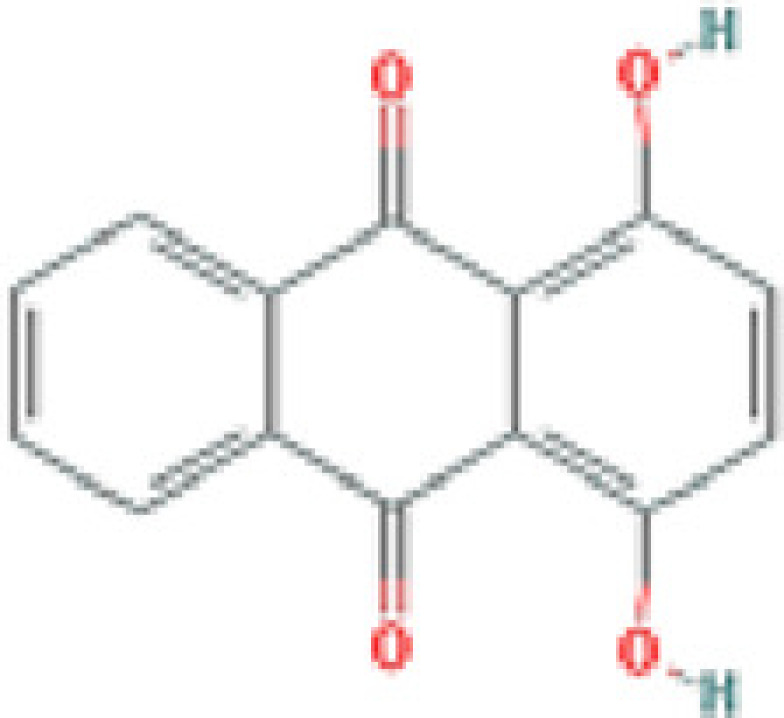	47.34	0.19
MOL006465	Rubrofusarin-6-β-gentiobioside	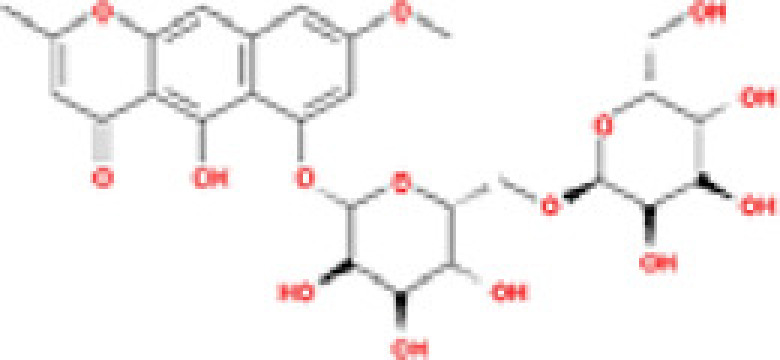	40.12	0.67

### Analysis of the drug-active compound-target-disease network diagram

**Figure 2:**
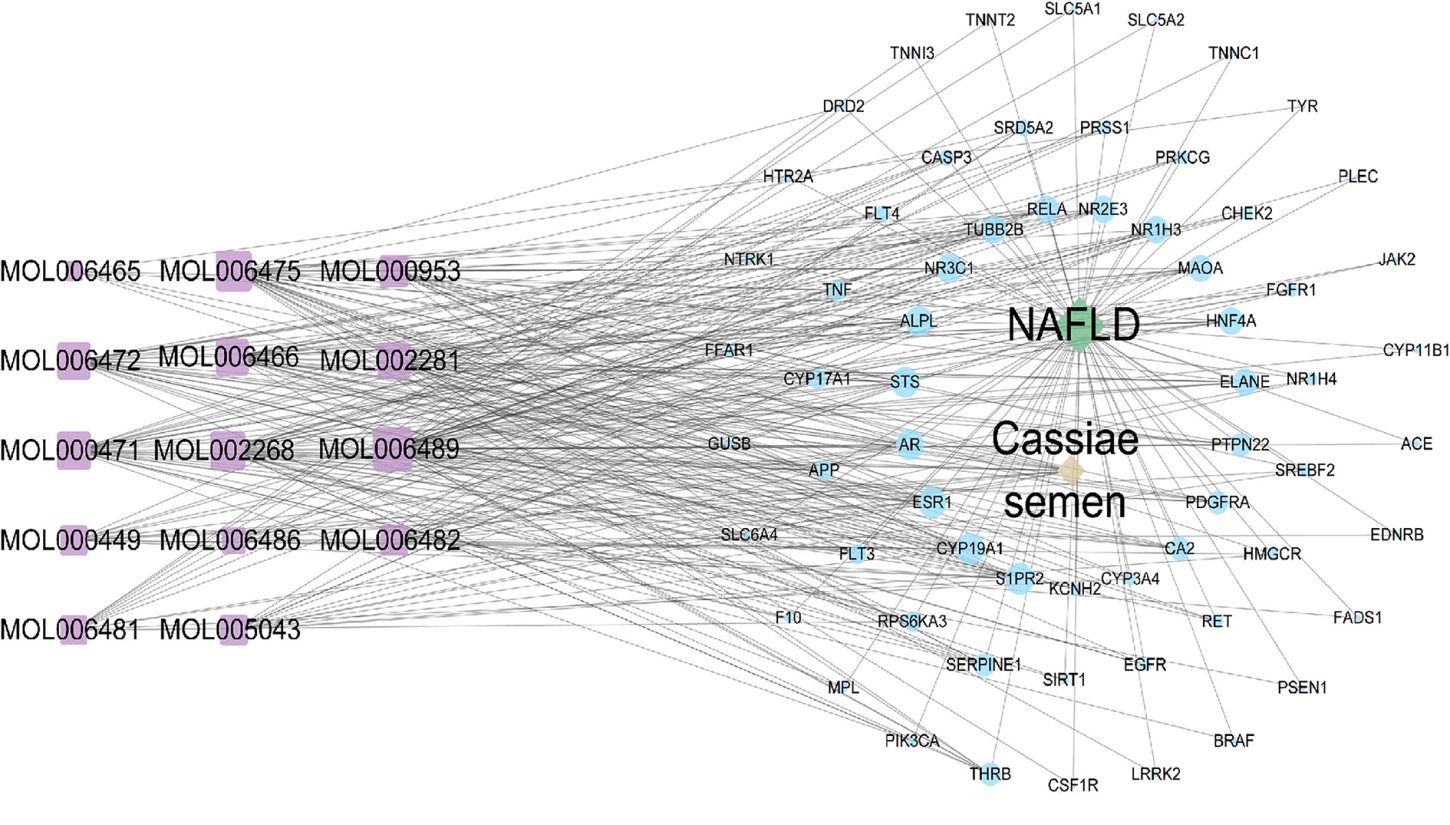
Drug-active compound-target-disease network diagram The blue circular represents 64 candidate genes of CS for the treatment of NAFLD, the purple rectangle represents 14 main components of CS, the green diamond represents NAFLD, and the yellow represents CS. The gray side represents the interaction between the targets, the size of the node represents the value of degree. The larger the node, the larger the degree.

The drug-active compound-target-disease network diagram established with the selected components and their targets are shown in Figure 2. The network included 80 nodes (14 CS components, 64 candidate genes, 1 disease name, and 1 drug name) and 341 edges. The purpose of the network diagram is to display complex compounds, multiple targets, and interactions between compounds and targets.

Seven components had higher degree values than the other compounds in the network: Quinizarin showed the highest degree of connectivity with 38 targets, followed by Obtusin with 31 targets, rhein with 29 targets, Aloe-emodin with 27 targets, Aurantio-obtusin with 26 targets, Rubrofusarin with 24 targets, and Toralactone with 24 targets, suggesting that these components were potential key active compounds in the treatment of NAFLD. Detailed information about the degree value is provided in the Supplementary Table S6.

### Analysis of the PPI network

**Figure 3:**
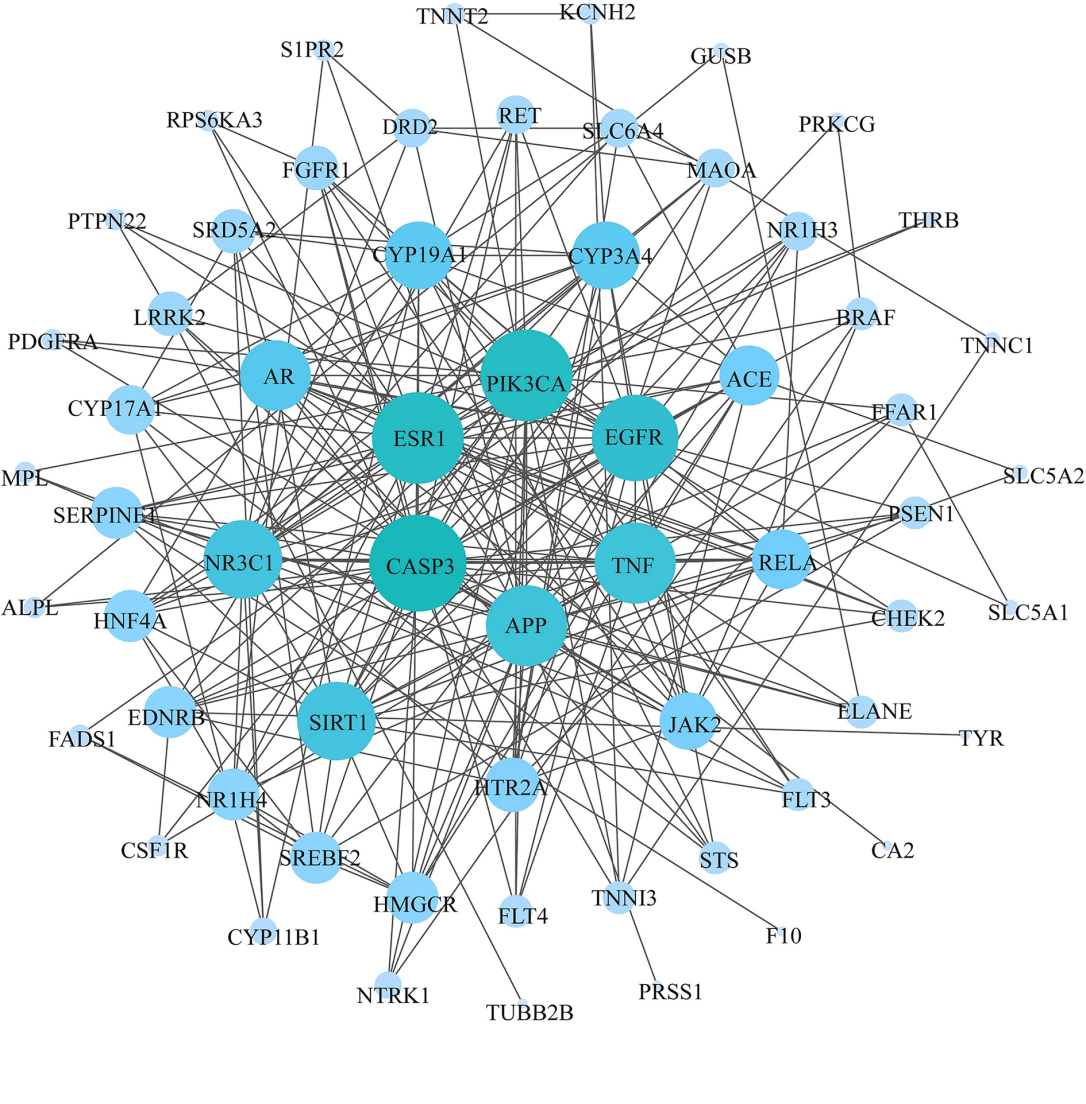
Protein interaction network diagram of candidate gene The node represents the candidate targets, the side represents the interaction between the targets, and the size and color of the node represent the value of degree. The larger the node, the larger the degree corresponding to the color from blue to ultramarine.

The 64 candidate genes obtained were integrated into the string database, after which the medium confidence > 0.4 and ‘Homo sapiens’ were selected. A total of 62 nodes and 249 edges were displayed using the Cytoscape software visualization, meaning that were 249 interactions in 62 candidate genes (Figure 3). Node size was positively associated with node degree. The genes with higher degree were considered as occupying the core positions in the PPI network and were more likely to serve as key core targets. Consequently, the targets including Caspase-3 (CASP3), estrogen receptor 1 (ESR1), phosphatidylinositol-4,5-bisphosphate 3-kinase, catalytic subunit α (PIK3CA), epidermal growth factor receptor (EGFR), tumor necrosis factor **(**TNF), and amyloidβ (A4) precursor protein (APP) played fundamental role in the treatment of NAFLD. The corresponding degree values are displayed in the Supplementary Table S7.

### Analysis of the KEGG pathway enrichment

**Figure 4:**
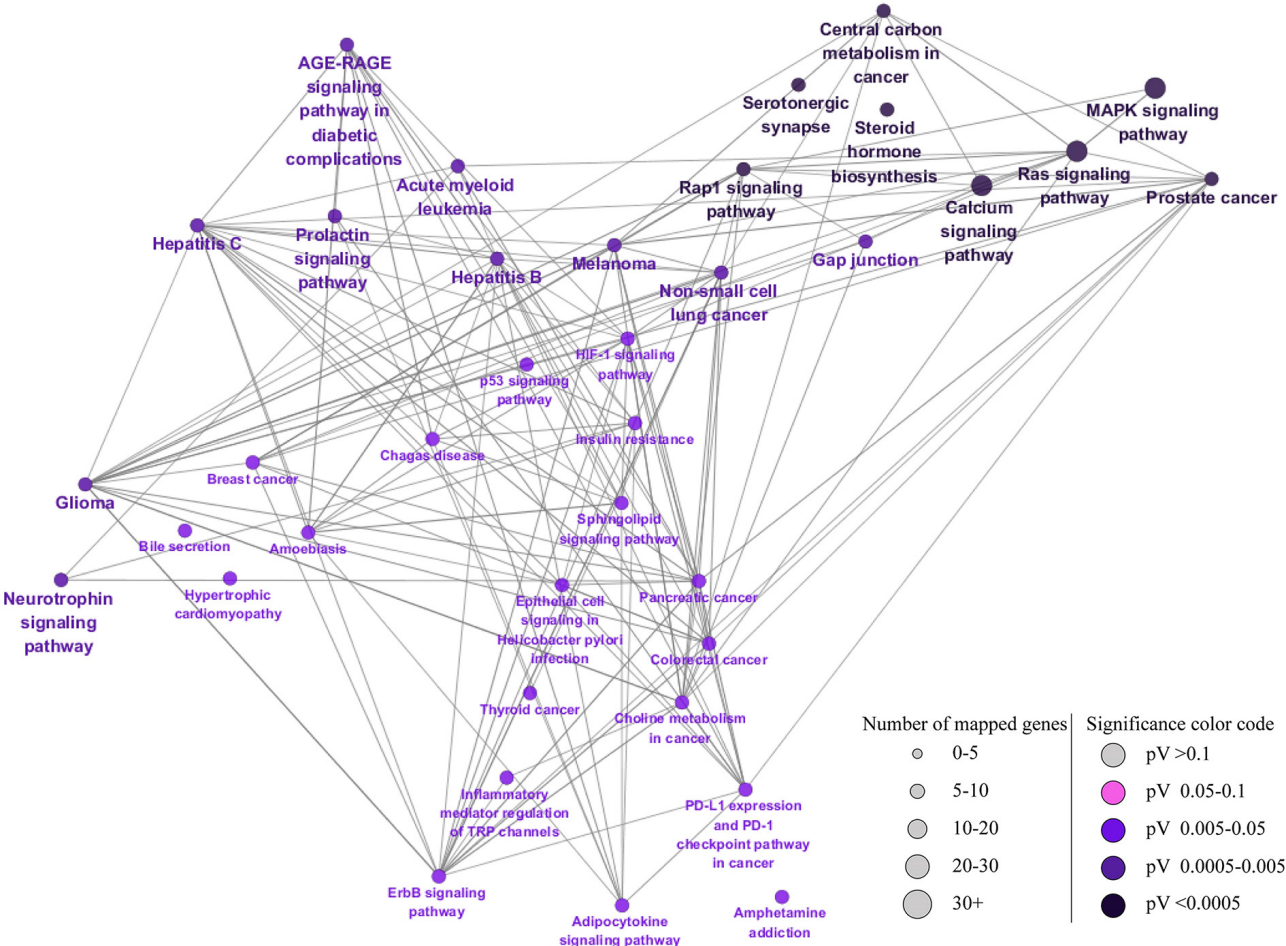
KEGG pathway analysis of the targets of CS for the treatment of NAFLD The size of the nodes reflects the enrichment significance of the terms. The more important the pathway, the smaller the *P*-value corresponding to the color from gray to black.

The KEGG pathway enrichment analysis identified 37 meaningful terms (*P*-value < 0.05), which are displayed in Figure 4. The top four enriched signaling pathways were Mitogen-activated protein kinase (MAPK), Ras, Ras-associated protein 1 (RAP1), and calclum signaling pathway, as determined using CLUE GO based on the number of mapped genes and the *P*-value of each term. Of the pathways, the MAPK signaling pathway was the most prevalent (*P*<0.0005) in each cluster, an indication that it was the most closely associated pathway with NAFLD and, hence, requires further evaluations.

### Analysis of the molecular docking

**Figure 5:**
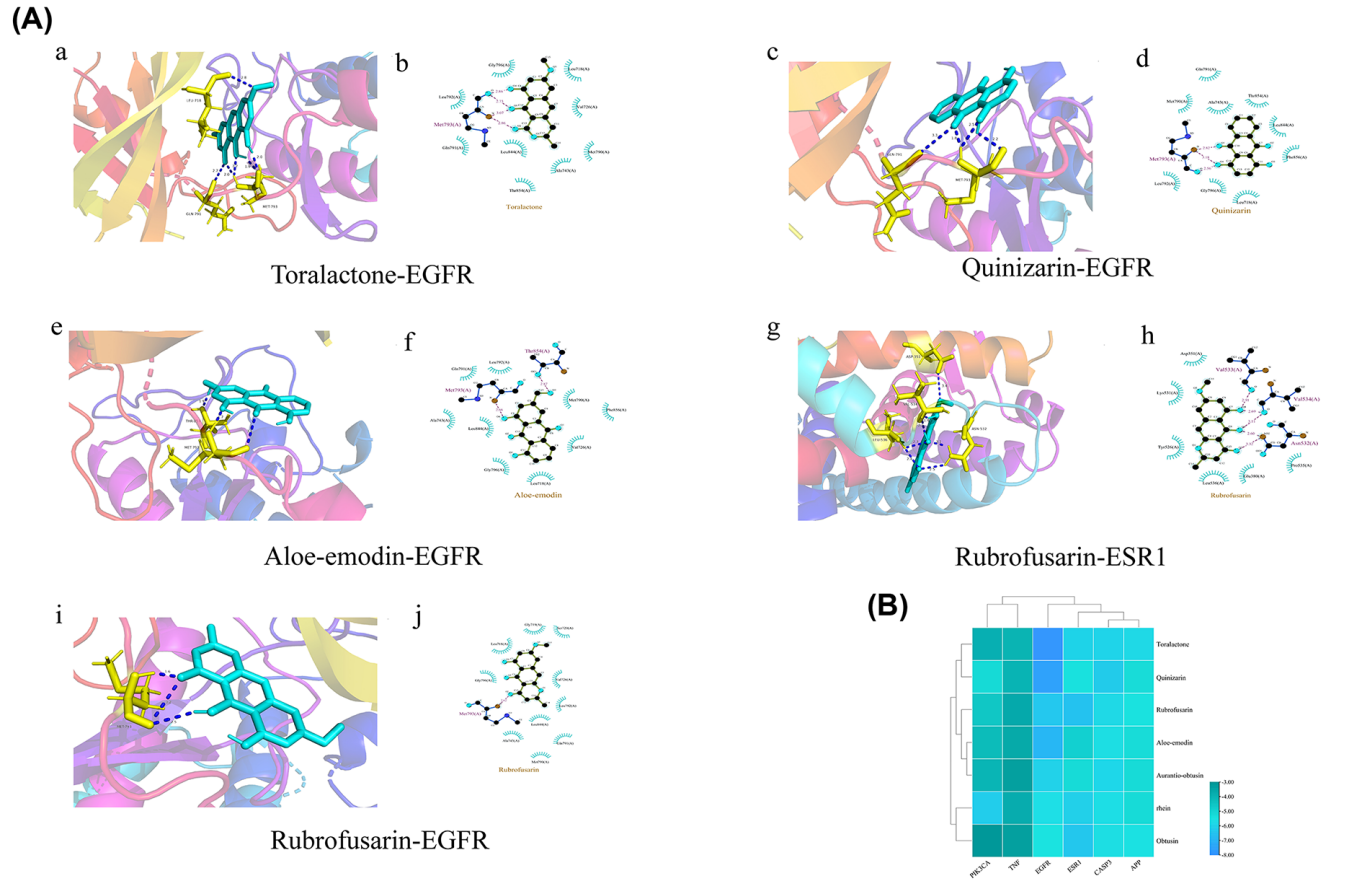
Molecular docking models of active compounds binding to potential targets (**A**) The visualization of the five combinations with the smallest binding energy. Panels a, c, e, g, and i represent the molecular model of the compound is in the binding pocket of the protein. Green represents the chemical structure of the component. Yellow represents the part of the target that binds to the component. Blue color dotted represents the conventional hydrogen bonds between drug and target. Panels b, d, f, h, and j show the interactions between compounds and surrounding residues. In the plan, purple color dotted line represents conventional hydrogen bonds. (**B**) Heat map of the binding energy of between the compound and target. *X*-axis: protein targets, *Y*-axis: active compounds. The blue the color, the greater the stability of the binding between the component and target.

The top 6 targets in the PPI network and the top 7 compounds of CS were docked. In the output of molecular docking, lower binding energy indicated that the binding force between the active ingredient and the target protein was strong. The binding energies are shown in Table 2. Binding energy less than 6 kJ·mol^−1^ pointed to an ideal binding affinity between the receptor and the ligand. The five visualized combinations with the smallest binding energy, including Toralactone-EGFR docking (−7.86 kcal/mol), Quinizarin-EGFR docking (−7.51 kcal/mol), Aloe emodin-EGFR docking (−6.92 kcal/mol), Rubrofusarin-ESR1 docking (−6.47 kcal/mol), and Rubrofusarin-EGFR docking (−6.36 kcal/mol) are shown in Figure 5 (the remaining visual combinations with binding energies less than 6 kca/mol^−1^ are provided in the Supplementary Figure S1). Of the combinations, the binding of EGFR with Toralactone (MOL002281) and with Quinizarin (MOL006489) produced the tightest binding energies.

**Table 2:** The binding energy of the main components of the docking to the core target

Mol ID	Molecule name	Degree	Target	Binding energy/kcal·mol
MOL006489	Quinizarin	38	CASP3	−6.16
			ESR1	−5.44
			PIK3CA	−5.18
			EGFR	−7.51
			TNF	−3.99
			APP	−5.29
MOL006475	Obtusin	31	CASP3	−5.58
			ESR1	−6.34
			PIK3CA	−3.06
			EGFR	−5.49
			TNF	−3.32
			APP	−5.52
MOL002268	rhein	29	CASP3	−5.65
			ESR1	−6.11
			PIK3CA	−6.18
			EGFR	−5.67
			TNF	−3.73
			APP	−5.23
MOL000471	Aloe-emodin	27	CASP3	−5.59
			ESR1	−4.84
			PIK3CA	−4.23
			EGFR	−6.92
			TNF	−3.66
			APP	−5.29
MOL006472	Aurantio-obtusin	26	CASP3	−5.84
			ESR1	−5.25
			PIK3CA	−4.01
			EGFR	−6.04
			TNF	−3.32
			APP	−5.23
MOL006466	Rubrofusarin	24	CASP3	−5.75
			ESR1	−6.47
			PIK3CA	−4.55
			EGFR	−6.36
			TNF	−3.65
			APP	−5.46
MOL002281	Toralactone	24	CASP3	−5.95
			ESR1	−5.93
			PIK3CA	−3.9
			EGFR	−7.86
			TNF	−3.92
			APP	−5.75

### Evaluation of CSEE regulating lipid metabolism *in vitro*

**Figure 6:**
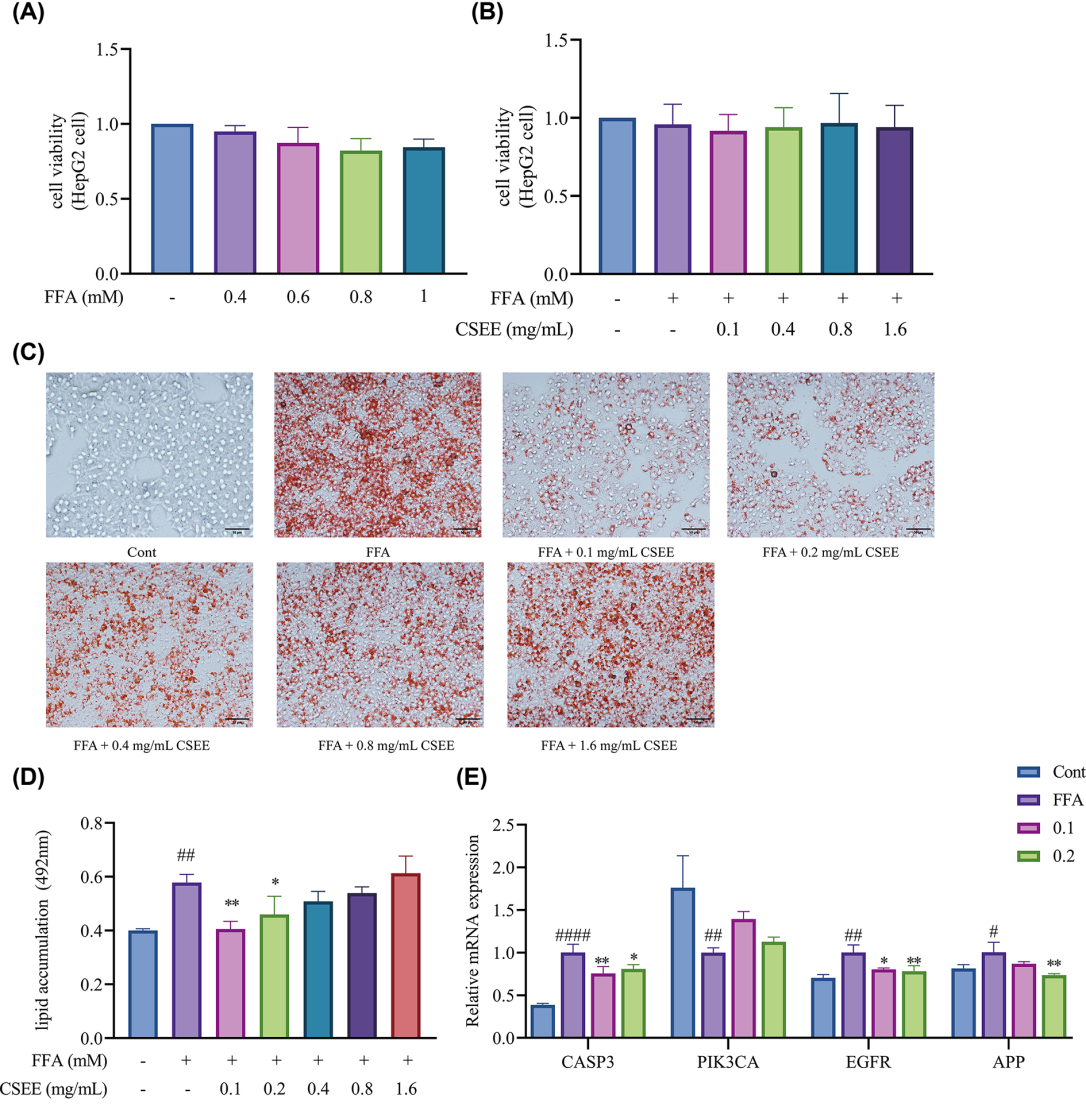
The effect of CSEE on lipid accumulation in HepG2 cell (**A**) Analysis of the cytotoxicity of HepG2 cell that were exposed to FFA (0.4–1 mM) for 24 h. (**B**) Analysis of the cytotoxicity of HepG2 cell pre-treatment with 1 mM FFA for 24 h and then treated with CSEE (0.1–1.6 mg/ml) for 24 h. Data were analyzed by one-way ANOVA with Tukey’s multiple comparisons test for the single variables. (**C**) Representative images of Oil red O staining of HepG2 cell. Photomicrographs are isolated from cells without special treatment (Cont), cells with FFA without CSEE treatment (FFA), cells with FFA and different concentrations of CSEE; scale bar: 50 μm, magnification: 200×. (**D**) Quantification of Oil red O staining. OD values were measured at an absorbance of 492 nm. (**E**) The expression of core targets in HepG2 cell pre-treatment with 1 mM FFA for 24 h and then treated with CSEE (0.1–0.2 mg/ml) for 24 h. Analysis of the effect of increasing concentrations of CSEE compared with untreated cells (FFA) was done by one-way ANOVA with Tukey’s multiple comparisons test. All the data of experiment represent means ± S.D. (*n*=3); ^####^*P*<0.0001, ^##^*P*<0.01 vs. Con; **P<0.01, *P<0.05 vs. FFA

The cytotoxicity of CSEE and 1 mM FFA on HepG2 cells was evaluated employing the CCK-8 assay. Based on previously published results [35–37] and the findings of the current experimentation (*P*>0.05, Figure 6A), a concentration of 1 mM FFA was considered the optimal modeling concentration for an *in vitro* model of NAFLD. The assay (Figure 6B) established no cytotoxicity after pre-treatment of HepG2 cells with 1 mM FFA for 24 h and then treatment with CSEE (0.1–1.6 mg/ml) for another 24 h, with cell viability above 80% for all tested concentrations (*P*>0.05).

To evaluate the effect of CSEE in regulating lipid metabolism using Oil Red ‘O’ staining, the 24-h FFA HepG2 cell model was treated with different concentrations of CSEE. Compared with the control group (0.400 ± 0.007), the 1 mM FFA model group (0.579 ± 0.031) without CSEE treatment had significantly increased lipid droplets (*P*<0.01). In contrast, lipid accumulation diminished in a concentration-dependent manner in the presence of the various concentrations of CSEE, the OD values were noted as 0.405 ± 0.029, 0.459 ± 0.069, 0.509 ± 0.037, 0.539 ± 0.024, and 0.613 ± 0.064 for the 0.1 (*P*<0.01 vs. FFA), 0.2 (*P*<0.05 vs. FFA), 0.4, 0.8, and 1.6 mg/ml CSEE concentrations, respectively. Remarkably, lipid droplets and lipid accumulation both substantially reduced after treatment with 0.1 mg/ml of CSEE compared with the FFA model group (*P*<0.01) (Figure 6C,D).

Following the establishment of the effective concentration range of CSEE after the analysis of Figure 6C,D, CSEE at concentrations of 0.1–0.2 mg/ml was selected for key target verification. Four key targets of CS for the treatment of NAFLD, including CASP3, PIK3CA, EGFR, and APP mRNA expression levels were validated using real-time PCR, revealing significantly up-regulated CASP3 (1.003 ± 0.0964 vs. 0.389 ± 0.017, *P*<0.0001), EGFR (1.003 ± 0.088 vs. 0.705 ± 0.038, *P*<0.001), and APP (1.004 ± 0.118 vs. 0.816 ± 0.043, *P*<0.05) gene expressions and down-regulated PIK3CA (1.001 ± 0.056 vs. 1.762 ± 0.374, *P*<0.01) gene expression in the model group compared with the control group. CSEE at a concentration of 0.1–0.2 mg/ml markedly down-regulated the gene expressions of CASP3, EGFR, and APP and up-regulated that of PIK3CA in the *in vitro* model of NAFLD. (Figure 6E). The details of the statistics are shown in Table 3.

**Table 3:** The statistics information of core targets for each group

Target	Cont	FFA	0.1	0.2
CASP3	0.389 ± 0.017	1.003 ± 0.096^####^	0.758 ± 0.078^**^	0.810 ± 0.049^*^
PIK3CA	1.762 ± 0.374	1.001 ± 0.056^##^	1.395 ± 0.087	1.128 ± 0.056
EGFR	0.705 ± 0.038	1.003 ± 0.088^##^	0.804 ± 0.015^*^	0.783 ± 0.064^**^
APP	0.816 ± 0.043	1.004 ± 0.118^#^	0.870 ± 0.026	0.7373 ± 0.016^**^

Note: Results represent mean ± S.D. (*n*=3). Significance: ^####^*P*<0.0001, ^##^*P*<0.01 vs. Cont; **P<0.01, *P<0.05 vs. FFA

## Discussion

There is growing clinical concern about NAFLD because of its high incidence and association with severe comorbidities, as well as the lack of effective treatment options for its [38,39]. CS has antihyperlipidemic and hepatoprotective effects, suggesting its role in the treatment of NAFLD [8]; however, few studies have examined the specific mechanisms underlying its effects on NAFLD. Based on network pharmacology and molecular docking, the present study explored the potential molecular mechanisms through which CSEE may treat of NAFLD.

In this investigation, seven active ingredients of CS were identified through drug-active compound-target-disease network diagram analyses and further examined. Our experimental results corroborate findings from previous literature [40–42] identifying the seven main ingredients of CSEE using liquid chromatography-mass spectrometry. Molecular docking established tight binding of toralactone, quinizarin, aloe-emodin, rubrofusarin, and aurantio-obtusin with the most important hinge region residue, MET-793 on EGFR. In addition, toralactone and quinizarin differentially bound EGFR to form three (MET-793, GLN-791, and LEU-718) and two (MET-793 and GLN-791) hydrogen bonds, respectively, suggesting that toralactone and quinizarin are the main active ingredients of CSEE in the treatment of NAFLD. Past reaearch outlined the antihyperlipidemic and hepatoprotective effects of toralactone and quinizarin including, toralactone’s protection against HepG2 cell death through antioxidative signaling [43], and quinzarin’s inhibition of adipogenesis and stimulation of lipolysis by down-regulating the sterol regulatory element binding the protein signaling pathway [44]. Nevertheless, further assessment is required to confirm these effects in an *in vitro* model of NAFLD and definitively determine the precise underlying mechanisms of these processes.

According to network pharmacology screening, CSEE may have direct or indirect effects on NAFLD through 64 potential targets, including CASP3, EGFR, PIK3CA, and APP. In our *in vitro* cell model of NAFLD, FFA overloading contributed to fat accumulation through the modulation of gene expression, but CSEE alleviated this fat accumulation by neutralizing the adverse changes in gene expression in response to FFA.

In the reversal of lipid accumulation in cell models of NAFLD using CSEE, low-concentration CSEE was the most effective. Also, high-dose treatment lessened the therapeutic effects, further supporting the efficacy of a low-dose. According to our real-time-PCR findings, treatment with low-CSEE concentrations (0.1–0.2 mg/ml) reversed the FFA-induced up-regulation of CASP3, EGFR, and APP genes and down-regulation of PIK3CA, indicating that CASP3, EGFR, PIK3CA, and APP are targets of CSEE and mediate its lipid-lowering properties. Of note, CASP3 was the most frequently identified molecule by the PPI analysis, pointing to its potentially critical role in NAFLD. CASP3, an intracellular cysteine protease, mediates apoptosis and inflammation through the processing and activation of pro-inflammatory cytokines. As one of the mediators of apoptosis, CASP3 can be regulated by multiple factors. Previous studies have shown that it modulates hepatocyte responses to toxic levels of lipids [45]. FFA-overload-induced lipotoxicity in hepatocytes results in activation of the caspase pathway, an important mediator of hepatocyte apoptosis [46,47]. Although there is no direct evidence that CASP3 regulates lipid metabolism, our scrutiny of the *in vitro* NAFLD model in this investigation established up-regulated CASP3 expression in NAFLD, which was reversed by treatment with CSEE. P38, a key factor in the MAPK pathway, is reportedly a possible actor upstream of caspases and a mediator of apoptosis in the hippocampus [48]. We suggest that CSEE affects the expression of CASP3 to improve the cellular metabolism of fat droplets by reducing the levels of hepatocyte apoptosis.

The high binding affinity between EGFR and the active ingredients of CS, as determined using molecular docking analyses, shows that EGFR is the most likely mediator of the therapeutic influence of CS on NAFLD. As a receptor tyrosine kinase of EGFR signaling, CS drives various cellular responses. Epidermal growth factor receptor signaling has been shown to have critical roles in all stages of liver responses to injury, from early inflammation and hepatocellular proliferation to fibrogenesis and neoplastic transformation [49]. Previous findings have posited that EGFR is in some way involved in lipid metabolism through the modulation of the expression of genes and the activities of enzymes participating in fatty acid synthesis and lipolysis [50]. In the present investigation, we found that FFA induced lipid accumulation by activating EGFR in HepG2 cells. Per Song et al., EGFR inhibition or EGFR expression silencing reversed the accumulation of lipid [51]. Other studies have shown that EGFR/MAPK signaling prevents the accumulation of lipids in liver cells by stimulating the phosphorylation of EGFR and MAPK [52]. Because traditional Chinese medicine always yields efficacy through multiple targets, CSEE can simultaneously act on APP and PIK3CA. A recent review reported that APP can be cleaved by proteases at two different sites to produce various short peptides, which can have either protective or negative effects on metabolism [53]. A previous study demonstrated a key role for PIK3CA in driving epithelial-to-mesenchymal transition and cancer stem cell phenotypes during cancer progression [54]. Hon et al demonstrated that different sites of somatic missense mutations in PIK3CA had differential effects on lipid kinase activities and cellular lipid binding levels [55]. CSEE's ability to induce PIK3CA gene expression in steatosis HepG2 cells remains unclear, but we suspect that the activation of phospholipid metabolic processes possibly contributes to this effect; still, further studies must be conducted to confirm this suspicion.

According to our KEGG pathway analyses using Cytoscape, the MAPK signaling pathway was the predominantly enriched pathway. MAPK has three main families: extracellular signal-regulated kinase, Jun amino terminal kinases, and stress-activated protein kinases. Inhibiting P38 and Jun N-terminal kinase, as demonstrated by one report, decreases hepatocyte steatosis and apoptosis in a rat model of NAFLD [56]. The MAPK signaling pathway plays an important role in the secretion and regulation of inflammatory cytokines in hepatic stellate cells [57]. Therefore, blocking the MAPK signaling pathway could result in significant hepatoprotective effects. Of note, our KEGG analyses established that CASP3 and EGFR were involved in the MAPK signaling pathway. CSEE also decreased lipid accumulation by reversing the FFA-induced elevation of CASP3 and EGFR gene expressions. In a nutshell, the MAPK signaling pathway possibly mediates the therapeutic effects of CSEE on NAFLD.

In summary, the present study made use of network pharmacology, molecular docking, and *in vitro* experiments to show that treatment of lipid metabolism with low CSEE concentrations significantly reduces lipid accumulation by modulating the MAPK signaling pathway to decrease CASP3 and EGFR expressions. This research provides theoretical and experimental bases for further investigations on the efficacy and mechanism of action of CS in treating NAFLD. However, these findings may have some limitations because the databases used conceivably do not include all known or unknown targets and protein–protein interactions associated with CS. Expanding these databases further could lead to a greater understanding of the effects of Cassiae semen on NAFLD, which would enable more comparative scrutiny of the composition of CS extracted using ethanol and alcohol in the next experiment so as to explore the effect of CS more fully on lipid metabolism.

## Supplementary Material

Supplementary Figure S1 and Tables S1-S7

## Data Availability

The data used to support the findings of this study are available from the corresponding author on reasonable request.

## Abbreviations

APP: Amyloidβ (A4) precursor protein
CASP3: Caspase-3
CS: Cassiae semen
CSEE: CS ethanol extract
EGFR: Epidermal growth factor receptor
ESR1: Estrogen receptor 1
NAFLD: Non-alcoholic fatty liver disease
PIK3CA: Phosphatidylinositol-4,5-bisphosphate 3-kinase, catalytic subunit α
PPI: Protein–protein interaction
TIC: Total ion chromatogram
TNF: Tumor necrosis factor
