# Supplementary material for: Exploring the mechanism of Cassiae semen in regulating lipid metabolism through network pharmacology and experimental validation
Source: Biosci Rep. 2023 Feb 7;43(2):BSR20221375. doi: 10.1042/BSR20221375 (PMC9905789; doi:10.1042/BSR20221375)
Supplement: Supplementary Figure S1 and Tables S1-S7 [file BSR-2022-1375_supp.pdf]

The supplementary data section contains 7 tables and 1 figure.

Table 1: The gene primers sequences

Gene name	NCBI reference sequence	Forward prime (5′ to 3′)	Reverse prime (5′ to 3′)
SDHA	NM 001294332.2	GTCCCTCCAATTAAACCAAACG	GTTCCGATGTTCTTATGCTTCC
CASP3	NM_001354777.2	CCAAAGATCATACATGGAAGCG	CTGAATGTTTCCCTGAGGTTTG
PIK3CA	NM_006218.4	CGGTGACTGTGTGGGACTTATTGAG	TGTAGTGTGTGGCTGTTGAACTGC
EGFR	NM_001346897.2	ACCCATATGTACCATCGATGTC	GAATTCGATGATCAACTCACGG
APP	NM_000484.4	CGTCACGTGTTCAATATGCTAA	TTCATGCGCTCATAAATCACAC

Table 2: The chemical composition of CSEE

Number	Name	Rt/min	Formula	Molecular Weight	ESI	MS^2 fragments
1	Choline	1.26	C5H13NO	130.10	$[M+H]^+$	104.1073, 60.0814
2	Dulcitol	1.27	C6H14O6	182.08	$[M-H]^-$	181.0711, 101.0234, 89.0234, 71.01277, 59.0128
3	3-methyl-5-oxo-5-(4-toluidino) pentanoic acid	1.28	C13H17NO3	257.10	$[M+H]^+$	258.1099, 124.9998, 104.1073
4	Raffinose	1.28	C18H32O16	504.17	$[M+FA-H]^-$	503.1621, 89.0234
5	Sucrose	1.30	C12H22O11	342.12	$[M+FA-H]^-$	341.1088, 179.0447, 89.0234, 71.01279, 59.01278
6	Trigonelline	1.30	C7H7NO2	137.05	$[M+H]^+$	138.055
7	D-Raffinose	1.31	C18H32O16	504.17	$[M+H]^+$	352.1128，163.0599，145.0495，127.039

						1
8	L-Valine	1.36	C5H11NO2	117.08	$[M+H]^{+}$	72.0814
9	Adenosine	1.70	C10H13N5O4	267.10	$[M+H]^{+}$	136.0617, 268.1037
10	4-Oxopyrrolidine-2-carboxylic acid	1.70	C5H7NO3	129.04	$[M-H]^{-}$	128.0344
11	Uridine	1.70	C9H12N2O6	244.07	$[M-H]^{-}$	110.0239, 200.0562, 243.0621
12	DL-Norleucine	1.86	C6H13NO2	131.09	$[M+H]^{+}$	89.0969, 69.0705
13	L-Phenylalanine	2.75	C9H11NO2	165.08	$[M+H]^{+}$	120.0801
14	2,3-Dihydroxybenzoic acid	3.60	C7H6O4	154.03	$[M-H]^{-}$	109.0286, 153.0186
15	Pentaethylene glycol	3.74	C10H22O6	238.14	$[M+Na]^{+}$	89.0602, 133.086, 239.1486
16	Indole-3-acrylic acid	4.20	C11H9NO2	187.06	$[M+H]^{+}$	146.06, 118.0654, 188.0706
17	Eriodictyol	5.12	C15H12O6	288.06	$[M+H]^{+}$	289.1255，179.0338
18	3,4-Dihydroxycinnamic acid	5.74	C9H8O4	180.04	$[M-H]^{-}$	135.0443, 179.0344
19	(-)-Fustin	6.99	C15H12O6	288.06	$[M+H]^{+}$	289.0704, 243.065

						1
20	Griseoxanthone C	8.89	C15H12O5	272.07	$[M+H]^+$	273.0755, 230.0573
21	Toralactone	8.90	C15H12O5	272.07	$[M+H]^+$	273.0755, 230.0572,
22	Aloe-emodin	9.02	C15H10O5	270.05	$[M+H]^+$	271.0598, 229.0494, 201.0545, 197.0596
23	Emodin	9.02	C15H10O5	270.05	$[M+H]^+$	271.0598
24	(2R)-5-Methoxy-2-methyl-2,3,8,9-tetrahydro-4H-furo[2,3-H] chromen-4-one	9.41	C13H14O4	234.09	$[M+H]^+$	235.0963, 217.086, 191.0701,
25	1-Dodecanesulfonic acid	9.41	C12H26O3S	250.16	$[M-H]^-$	249.153, 79.9564
26	Formononetin	10.64	C16H12O4	268.07	$[M+H]^+$	269.0806, 254.0571, 226.0623
27	Cassiaside B2	11.80	C39H52O25	920.28	$[M-H]^-$	271.0612, 256.0377
28	Maltotetraose	11.82	C24H42O21	666.22	$[M+H]^+$	85.0289, 145.0494, 127.039
29	Norlichexanthone	13.43	C14H10O5	258.05	$[M+H]^+$	259.0599, 213.054

						3, 241.0494
30	Fisetin	14.02	$C_{15}H_{10}O_6$	286.05	$[M+H]^+$	287.0548
31	Cynaroside	14.05	$C_{21}H_{20}O_{11}$	448.10	$[M-H]^-$	447.0932, 284.0326, 256.0376,
32	Aurantio-obtusin β-D-glucoside	14.18	$C_{23}H_{24}O_{12}$	492.13	$[M-H]^-$	491.1195, 476.0961, 313.0354, 242.0219
33	Rubrofusarin	16.74	$C_{15}H_{12}O_5$	272.07	$[M+H]^+$	273.0754, 255.0648, 230.0571, 227.0699, 199.0751
34	Isorhamnetin	17.79	$C_{16}H_{12}O_7$	316.06	$[M+H]^+$	317.0655, 274.047, 302.0420
35	Kaempferol	19.65	$C_{15}H_{10}O_6$	286.05	$[M+H]^+$	287.0547
36	1H-Naphtho(2,3-c) pyran-1-one, 3,4-dihydro-9,10-dihydroxy-7-methoxy-3-methylene-	21.17	$C_{15}H_{12}O_5$	272.07	$[M+H]^+$	273.0755, 255.0649, 227.07
37	Apigetrin	21.37	$C_{21}H_{20}O_{10}$	432.11	$[M-H]^-$	269.0456

38	Gluco-obtusifolin	21.67	$C_{22}H_{22}O_{10}$	446.12	$[M+H]^+$	285.0754, 270.052
39	Rhein	24.34	$C_{15}H_8O_6$	285.04	$[M-H]^-$	283.06, 268.04, 240.04
40	Diosmetin	25.53	$C_{16}H_{12}O_6$	300.06	$[M+H]^+$	301.0706, 286.0472, 258.0521
41	Glycitein	26.69	$C_{16}H_{12}O_5$	284.07	$[M-H]^-$	285.0756, 270.0521, 242.0572
42	Hispidulin	26.89	$C_{16}H_{12}O_6$	300.06	$[M-H]^-$	299.0563, 284.0328, 256.0376
43	Aurantio-obtusin	26.95	$C_{17}H_{14}O_7$	330.07	$[M+H]^+$	331.0814, 316.0576, 298.0473,
44	Alternariol	27.53	$C_{14}H_{10}O_5$	258.05	$[M+H]^+$	259.06, 213.0545, 241.0494
45	3-Methoxy-5,7,3',4'-tetrahydroxy-flavone	27.73	$C_{16}H_{12}O_7$	316.06	$[M-H]^-$	315.0510, 300.0275, 272.0326, 216.0425

46	Luteolin	27.95	C15H10O6	286.05	[M-H] -	285.0405，241.0505
47	Gardenin B	29.78	C19H18O7	358.11	[M+H] +	359.1123, 326.0785
48	Stigmasterol	33.71	C29H48O	411.36	[M+H] +	411.20, 347.35
49	Genistein	34.32	C15H10O5	270.05	[M-H] -	269.0457, 225.0554
50	Aurapten	35.49	C19H22O3	298.16	[M-H] -	297.1530, 183.0116
51	Quinizarin	36.36	C14H8O4	241.05	[M+H] +	241.12, 213.13, 69.03, 91.05
52	Cholesterol	38.60	C27H46O	387.36	[M-H] -	387.16, 355.13, 313.08
53	4-Dodecylbenzenesulfonic acid	39.16	C18H30O3S	326.19	[M-H] -	325.1843, 183.0116
54	Palmitoyl ethanolamide	39.56	C18H37NO2	299.28	[M+H] +	300.2899, 62.0607
55	N, N-Diethyldodecanamide	40.89	C16H33NO	255.26	[M+H] +	256.2632
56	Stearamide	41.78	C18H37NO	283.29	[M+H] +	284.2947

Table 3: Confluence of component targets

ABCG2	CES1	Grin1	PIK3CG	RARG	ERN1	Drd4	PGR	CYP3A4	SYK	BCHE
ADORA1	CES2	Grm2	PIM1	RELA	BCL2	FDPS	PSENEN	Ednra	GPBAR1	P2RX3
AHR	CHEK2	HCAR2	PLA2G1B	RIPK2	ABCB1	FFAR1	PTK2	PDE3A	OPRK1	FDFT1
AKR1B1	CSNK2A1	HNF4A	PLAT	RORA	ACE	FGFR1	S1PR1	TOP1	SRD5A1	IGF1R
AKR1C3	CTDSP1	HSD11B1	PLAU	RPS6KA3	ADRA1D	HDAC2	S1PR4	TYK2	SRD5A2	ALDH3A1
ALOX15	CTSG	HSD17B1	PLIN1	RXRB	ADRA2A	HDAC4	SIGMAR1	LIMK1	NPC1L1	EDNRB
ALOX5	CYP1A2	HSD17B2	PLIN5	RXRG	ADRA2B	HDAC6	SIRT2	LCK	HMGCR	DPP7
ALPL	CYP2C9	HSD17B3	PPO2	S1PR2	ANPEP	HSP90AB1	SLC6A2	NR4A1	CYP51A1	DPP8
APOBEC3G	Cnr2	HSP90AA1	PRKACA	SERPINE1	APH1A	HTR1A	SLC6A4	Adra1a	RORC	DPP9
APP	DHODH	HTR1E	PRKCB	SIRT1	APH1B	HTR2A	Slc6a3	CSF1R	SHBG	IL2
Adra2c	DNMT1	Htr2c	PRKCE	SLC9A1	APOBEC3A	HTR5A	TAAR1	EPHB4	SREBF2	ALDH2
BCL2A1	DRD1	Htr6	PRKCG	STS	AR	Hrh1	TGM2	FLT4	PDE4A	LGALS9
CA1	DRD5	IKBKB	PRSS1	TBXA2R	CACNA1H	Htr1b	TNF	KCNH2	S1PR3	TYR
CA12	DUSP3	KIF11	PRSS2	TERT	CDK1	Htr3a	EGFR	LRRK2	BRAF	Hrh3
CA13	Dyrk1a	MAOA	PTGER1	THRA	CHRM1	Htr7	PLEC	MAPK14	PSEN1	QPCT
CA14	ELANE	MAOB	PTGER2	THRB	CHRM2	IMPDH2	CSNK1A1	NTRK1	PSEN2	JAK3
CA2	ESR1	MAPK10	PTGER4	TLR9	CHRM3	JAK2	CSNK1D	RET	NR1H4	DYRK1B
CA4	ESR2	MCL1	PTGES	TUBA1A	CHRM4	KCNA3	TNNC1	RPS6KB1	GUSB	HDAC1
CA5A	F10	MGLL	PTGFR	TUBB2B	CHRM5	KDR	ADORA3	ZAP70	FNTB	FLT1
CA5B	F3	MIF	PTGS1	UL80	CHRNA4	MDM2	SLC5A2	PLK1	TNNT2	CCNB3
CA6	FLT3	MMP12	PTGS2	WEE1	CLK1	MMP2	SLC5A1	AURKB	TNNI3	CDK5R1
CA7	FOLH1	MPL	PTPN1	XDH	CNR1	MMP9	EPHX2	CYP1B1	CCNB1	CCR4
CA9	FYN	NR1H3	PTPN2	ache	CXCR2	Mme	SLC29A1	CDK6	CCNB2	CDC42
CACNA1B	GALR3	NR2E3	PTPN22	lef	CYP11B1	NCSTN	ADORA2B	CBR1	FNTA	PDE4B
CASP3	GPR35	NR3C2	PTPN7	FTO	CYP11B2	NOS3	IGFBP3	PTPRS	FADS1	GSK3B

CASP9	GRIA2	Nos2	PTPRC	CYP19A1	CYP17A1	NPY5R	IMPDH1	DRD2	PARP1	LGALS3
CDC25B	GRIN2B	PDE4D	Pdgfrb	FNTA	CYP2C19	NR2F2	SLC5A4	PIK3CA	RARB	LDHB
CDK2	GRM4	PDE7A	RAC1	PTP4A3	CYP2D6	NR3C1	ADK	CDK5		
CDK4	GSK3A	PDGFRA	RARA	LDHA	Cckar	Nos1	LGALS4			

Table 4: Confluence of disease targets

MANBA	ATP7A	BRCA2	DYSF	KIF21A	CLN8	ANO5	SLC1A1	EPHA2	ITGA7	COQ8A	EIF2B4	CTSC	TIMM8A	GALT	STS	ADAMTSL4
MC4R	POLG	STK11	CCM2	OPN1LW	WHRN	FGF9	TULP1	ERCC6	ITPR2	RPGRIP1	SLC25A46	SEPTIN9	ASXL1	SAMHD1	MAPT	CTSA
MECP2	ATRX	SURF1	SLC4A11	RPGR	GAMT	GM2A	ZIC3	LRTOMT	JUP	IFT80	NEXN	SRCAP	DLD	AUTS2	MAX	TET2
MEFV	UGT1A1	BTD	PLA2G6	EMG1	HPD	HOXA1	BSND	FCGR2B	KISS1	PTS	LARGE1	ADAMTS13	DLG3	GATA2	CHST6	APTX
MET	PPP1R3A	TBX1	PHF6	FGD4	HPS1	HSPD1	DYNC2H1	FGFR1	PCARE	RIT1	LRAT	GLMN	DLX3	SACS	KMT2A	BCOR
ASAH1	AVP	TBX5	CASR	NKX2-5	PFKM	KCNH2	CEP290	CILK1	LIN28B	RLBP1	REEP6	WDR45	DMP1	GCH1	TRPM1	MKS1
MITF	FERMT1	TCF4	IKBKG	DSP	POLH	LAMB3	CLPP	KIF1B	LIM2	RP1	S1PR2	CHEK2	DNMT3A	CNNM4	MPO	SLC29A3
MPL	SLC39A4	TBX3	ELP1	AKT2	BBS1	MGAT2	MKKS	SATB2	MEN1	SALL1	ACVR2B	PRRT2	DPYD	IL36RN	MRE11	TMEM127
MPZ	CHD7	TCOF1	SHANK3	EVC	SCO1	NEB	LZTR1	RPGRIP1L	MID1	ATXN2	RAB28	KRT71	DRD2	GFPT1	GPR179	PRKCG
ASPA	PRKAR1A	BTK	RUNX2	KIFBP	SGCD	MYO15A	KDM5C	CLDN14	MIP	SCN1A	BAG3	TMC6	DSG1	AMH	MTHFR	MAP2K2
MSH2	MAP2K1	BUB1B	TP63	NPHP3	NMNAT1	SERPINF2	FOXN1	ARL2BP	MLH1	SCN8A	SLC12A6	SLC46A1	AGXT	ATP2C1	MTM1	PROC
TRIM37	PRNP	TFR2	HESX1	INVS	TRPS1	CLN6	SERPINH1	LRRC6	MMP1	SCNN1B	AMHR2	SLC52A3	ECM1	GK	MVK	PRSS1
MYC	SLURP1	TGFBR1	CCN6	IHH	WNT7A	PRF1	LRSAM1	FXN	ND6	VIPAS39	FASLG	CHRND	EDA	DISC1	MYH3	HTRA1
SERPINC1	SALL4	TGFBR2	KRIT1	LRP2	ADGRV1	WDR35	TRIP11	G6PC	MMUT	SHH	HPSE2	CCDC151	EIF2B1	GLB1	MYH7	FAM20C
MYH9	SELENON	TGM1	MTMR2	PNP	CASP8	NYX	BCKDK	NIPBL	MYBPC3	PRSS56	ALX4	SLC22A12	ELANE	GLRB	NEUROD1	PNPLA2
MYO5B	PTCH1	TIMP3	SGCE	RPE65	AP3B1	SIX3	TCIRG1	GALNS	MYH6	SIX1	SGSH	ERCC8	ELN	AMT	ATP1A2	MCOLN1
MYO7A	ARID1B	TNFRSF1A	PHOX2B	SGCB	SLC25A12	REEP1	TUBB3	CNTNAP2	MYO5A	SLC4A1	TGFB2	ANTXR2	EMD	GNA11	NGF	JPH3
NBN	PTPN11	TSC2	ACVR1	TNNI2	AGPAT2	PCDH15	POMT1	ABCA12	MYO6	SLC12A3	OFD1	CLCNKB	C9orf72	GNAQ	NOTCH3	TBC1D24
NDP	BARD1	TTN	LGI1	FKRP	CHKB	SLC12A1	CLDN16	GARS1	ATP1A3	SNCA	DLL3	TPH2	EPOR	GP9	NPHP1	ALS2
ATM	PYGM	TTPA	RECQL4	SPG11	CLCN7	GNPAT	LDB3	NPHP4	CISD2	ELOVL4	CPS1	TTC8	ERBB2	OSTM1	NPHS1	CHD8
NDUFS4	RAF1	TTR	ZEB2	OTOF	LRRK2	PAPSS2	PDCD10	PTPN22	PJVK	ABCC8	FLNB	EDARADD	ETFDH	NR3C1	ATP2A1	EPG5
NF1	RAG2	TWIST1	FIG4	KERA	CTSD	ENAM	TREX1	GAS8	DUOX2	TACR3	PDE6C	COL4A3	ALAS2	ANKRD11	NRAS	NLRC4
NF2	RET	UBE3A	AMER1	COL1A2	FGF10	CLP1	CHRNB2	GATA1	CDON	TBP	UNC13D	COL4A4	ETV6	MSH6	NTRK1	RAB27A
NHS	RPS6KA3	UMOD	COCH	COL10A1	FAN1	CHAT	CHRNE	GATA4	TBX22	TBX6	MSX2	COL6A2	EYA1	ICOS	GPR143	RAG1
NOTCH2	RYR1	UROS	LIG4	C8orf37	FOXE1	TMC1	CLCN1	TINF2	SOST	TECTA	NAGLU	SLC6A4	RUNX1	COX7A2	SPAT	HKLLS2

NPC1	RYR2	USH2A	EPCAM	DCN	SLC35D1	DAG1	CNTN1	GDI1	ZDHHC9	THRB	WNT5A	SMARCA2	CBL	SEC63	HEDJ	LFIS
ATP2A2	ATXN1	CLRN1	ANKH	FANCC	DDHD2	LCA5	COL4A1	GJA3	CD320	TNNC1	MAGI2	SMARCB1	TNFRSF11A	FABP7	LCE	SCA45
ROR2	SCN4A	VHL	SMOC1	FGF14	CRB1	EDNRA	COL4A5	DNAI1	ZMYND10	TNNI3	CSF3R	SMPD1	PROM1	RINT1	VMLDS2	SCA38
OAT	SCN5A	BEST1	TFAP2B	ZFYVE26	AIPL1	PHF8	KLHL40	GJB3	PDE6A	TNNT2	STRC	SMS	PER2	FABP5	FAT	STGD2
SMARCAL1	BLM	VWF	CA2	MMACHC	GJA8	FLNC	COX6A1	GNMT	PDE6B	TPI1	EPB41	SNRPN	EIF2B2	FABP12	LARS	BLBP
PRKN	NOD2	WAS	EIF2B5	PRPF31	HSPG2	AMACR	CP	GNRH1	PDHA1	TPO	CAVIN1	SPTA1	BCL10	CDH17	MEGF1	EFABP
PAX2	ABCG8	WRN	CLCN5	KRT5	KIF1A	SLC17A8	CREBBP	PNPLA1	SERPINF1	TRPC6	P3H1	SRD5A2	PSTPIP1	SLC27A4	ILLBP	HPT1
PAX3	SGCA	WT1	COL11A1	KRT16	CNGB3	MSRB3	CRX	ILDR1	PHKA1	TSC1	STX11	SRY	SLC7A7	FFAR4	SSC2	IPS
PAX4	SGCG	XPA	DDC	NEFL	PPIB	GNAO1	CRYAA	GRID2	POMC	TSHR	HPS5	STAR	USP8	ELOVL3	SPDA1	GPR120
PAX6	SH3BP2	XPC	F13A1	NPR2	GJC2	DOK7	CRYAB	GRM1	MAGEL2	TYR	CRYGC	STAT3	SLC6A5	FAR1	HELO1	PFCRD
FOXP3	PORCN	CACNA1A	GRN	SERPINI1	ACTA1	ANK1	CRYGD	CFH	LZTFL1	UROD	MEGF8	STIM1	NOG	PURAQTL1	ADMD	LRP7
ABHD5	SLC3A1	CACNA1C	KIF7	RP2	PINK1	GRM6	CRYGS	HSD17B4	AHI1	VCP	LRP4	TAF1	BBIP1	FADS1	PCLD2	PCLD3
SBDS	SLC6A8	CACNA1F	KRT14	CDH23	SOX10	HMX1	HJV	HYAL1	PPOX	NSD2	DYM	TAT	DRC1	FADS2	FABPB	MTRNS
PDGFRB	SLC16A2	ALMS1	PEX7	TNNT1	SPG7	OTOG	CYBB	CCDC39	ARMC4	ZIC2	LMBR1	TAZ	ADGRG1	FADS3	ILFS3	EDN
SUFU	BMPR1A	CXCR4	THAP1	FA2H	GNPTAB	KCNJ1	ACE	FREM2	SLC35C1	CA8	HPS3	HNF1B	MMP20	ALG8	PAFABP	VLACS
SLC26A4	SLC22A5	EPM2A	PPT1	CASQ2	FERMT3	MAOA	GLIS3	IDUA	AVPR2	BBS10	MARVELD2	ACTC1	EFTUD2	FAT3	CDH16	ALR
PHEX	SMN1	SH3TC2	WFS1	SERPINA6	AP1S2	MATN3	DKC1	TUBB2B	DHTKD1	OPA3	DZIP1L	TFAP2A	GRHPR	GYS2	FATP4	FAXDC1
PHYH	SOD1	EHMT1	NDRG1	TMEM67	PRPF3	CRBN	DNAH5	IGF2	POMGNT1	CSRP3	FLNA	TGFBI	FOXP2	FAR2	O3FAR1	SLS
PKD1	SOS1	L2HGDH	PARK7	SNAP29	IFT140	PITX2	DNASE1	APP	VPS35	PABPN1	BLOC1S6	NKX2-1	RP1L1	GLS2	CIG30	PCLD1
PKD2	SOX9	FRAS1	CLN5	ECEL1	CRTAP	GDAP1	DYNC1H1	DNAAF3	TDP1	GAN	GNAT2	TPMT	ITM2B	RNASE2	MLSTD2	VLACSR
PKLR	FOXL2	SLC19A3	RNF168	STUB1	CIB2	BBS2	DNM2	IGSF1	SCN3B	KISS1R	TPRN	U2AF1	SCARB2	FITM1	BMND1	SIDDIS
PKHD1	SPAST	FGF23	F12	CNGA3	PRPF8	BBS4	JAG1	AQP2	HR	AGPS	RD3	KDM6A	BMS1	PYGL	GLIS2	HULC
PLEC	SPTBN2	KMT2D	MFSD8	COX10	CHUK	PRPH2	SLC26A2	INSL3	CFC1	CAV3	BLOC1S3	VIM	PLEKHM1	SLC27A2	TCAP	ELOVL2
PLP1	BRCA1	GDF5	ABHD12	CTH	USH1G	RHO	TOR1A	PDX1	LMOD3	DNAH11	LAMC2	DEE38	SOPH	SCA34	CAV1	HLA-B
PMP22	BRAF	CAPN3	PITX1	EGR2	DNMT3B	DCLRE1C	EDN3	ITGA2B	PSEN1	CBS	LTBP3	NAG	LDLCQ4	VBCH2	TNFSF11	ELOVL5
MUSK	HGSNAT	EZH2	HBA1	OCA2	RASA1	CACNA1S	MED12	MFGE8	GA	SPGFY2	SLC9A6	TRPV3	SLC37A4	FLDB	DJ9	CMT2U

MYO3A	TRPM6	F10	HBA2	OCRL	RB1	RAB7A	CDH1	GFER	MARS	ILFS2	SEC23B	DCTN1	WDR72	MTPA	FACE	SPG35
RS1	CRYBA1	FECH	HBB	OPA1	BCKDHA	VKORC1	FABP3	PHKG2	RNS2	FCHL2	SPTLC1	TMPRSS6	GALK1	OPTA1	ACAT1	PEX1
CARD14	CRYBB2	ALDH3A2	HCCS	TNFRSF11B	TRPV4	ALDH5A1	ELOVL1	PHKB	FIT1	ABBP2	CETP	DDX11	GALNT3	ZMPSTE24	CYP21A2	KAT6B
FAM161A	CRYGB	FGD1	HINT1	ALDH7A1	BCKDHB	CDC73	FAAH	PRKCSH	FACVL1	ILFS1	LAMA3	PLG	SLC5A1	CDKN1C	CYP27A1	LEMD3
ATCAY	SLC34A3	FH	MNX1	PAFAH1B1	RDH5	USB1	FAAHP1	FFAR1	ERV1	STGD3	LHCGR	PMM2	SLC5A2	CDKN2A	CYP27B1	FSHR
ADAM9	CSF1R	RAB3GAP1	HLA-DQB1	PAH	GRK1	PALB2	SLC27A3	FFAR3	GSD9C	PGR4	TACSTD2	PMS2	CPLANE1	ADAR	DNAAF4	ABL1
MFN2	OTOA	RAB18	HLCS	PAX7	ATXN7	PANK2	FMO1	FFAR2	ALDH10	LR3	MAF	ATR	SLC6A3	HAX1	SPRED1	FTL
ADA	DSG4	FOXC2	HSD11B2	PCCA	SCN9A	DCAF17	FMO4	SLC27A5	G19P1	METRS	KRT2	ATP8B1	SHOX	TTC25	LEAP2	SSC1
GNE	CTNNA1	SETX	HSF4	PCCB	SCN10A	CFAP43	ARV1	FIT2	GPR40	MPMCD	KRT9	ABCB4	P2RY12	BRIP1	LARS1	FAAHOUT
ABCC9	CTSK	FOXO1	HTR2A	SAR1B	SDHB	CUBN	NBAS	PFKL	GPR41	FAH1	KRT10	PIK3CD	SLC2A1	DTNBP1	FAT2	FATP3
USH1C	CYLD	SPART	LRIT3	PCNT	SDHC	SHOC2	LCO	TRMU	GPR43	FALDH	IFITM5	PIK3R1	SLC2A2	ZNF469	FABP6	PK1
PQBP1	ESCO2	FLT4	IL2RG	PDCD1	SDHD	AAAS	DNAJB11	SLC25A6	FATP5	VLCSH2	SIL1	BAP1	ELOVL7	HOPS	DFFRX	BMIQ10
OPTN	VPS13B	FMO3	IVD	PRKAG2	TRAPPC2	ACTN4	ELOVL6	PHKA2	C20orf142	PYKL	DFFRY	OPPG	TNSALP	ILLD	IKSHD	SCS7
SLC25A15	CYP11B1	DICER1	AGRN	CABP2	STRA6	SLC2A10	FABP2	USP9X	MTO2	MRXS99F	LINC00505	GSD9A	FAT1	USP9Y	PHK	ISQMR
PRG4	CYP17A1	TARDBP	KCNJ2	PDGFRA	ROBO3	CALR	FAT4	FAAH2	ANT3	PKD6	CYP19A1	SF3B1	KCNQ3	ATP8A2	ABCG5	AXIN1
SF3B4	EVR4	PCLD4	CDG1H	XLG	MLSTD1	MRX99										

Table 5: Intersecting target

Drug and disease intersection target	TNF	CYP3A4	TNNI3	DRD2	PRKCG
	NR1H4	ACE	TNNT2	ELANE	PRSS1
	NR1H3	FGFR1	TYR	F10	SLC5A1
	HNF4A	PTPN22	S1PR2	FLT4	SLC5A2
	RELA	TUBB2B	CHEK2	NR3C1	SLC6A4
	SIRT1	APP	CSF1R	HTR2A	SRD5A2
	SREBF2	PSEN1	CYP11B1	STS	FADS1
	CASP3	THRB	CYP17A1	NTRK1	FFAR1
	SERPINE1	TNNC1	CYP19A1	PDGFRA	

Table 6: The degree value of components in the Drug-active compound-target-disease network diagram

Copmoents name	Mol ID	Degree
Quinizarin	MOL006489	38
Obtusin	MOL006475	31
rhein	MOL002268	29
Aloe-emodin	MOL000471	27
Aurantio-obtusin	MOL006472	26
Rubrofusarin	MOL006466	24
Toralactone	MOL002281	24
Cholesterol	MOL000953	15
Campesterol	MOL005043	13
1H-Naphtho(2,3-c)pyran-1-one, 3,4-dihydro-9,10-dihydroxy-7-methoxy-3-methylene-	MOL006482	13
Stigmasterol	MOL000449	12
gluco-obtusifolin	MOL006481	11
obtusin	MOL006486	8
Rubrofusarin-6-beta-gentiobioside	MOL006465	6

Table 7: The degree value of the target where the disease and the drug intersect

Degree	Target	Degree	Target	Degree	Target	Degree	Target	Degree	Target	Degree	Target
26	CASP3	15	CYP19A1	9	NR1H4	5	TNNI3	4	CYP11B1	3	TNNT2
24	ESR1	12	ACE	8	CYP17A1	5	FFAR1	3	ALPL	2	SLC5A2
24	PIK3CA	12	RELA	7	LRRK2	5	ELANE	3	S1PR2	2	GUSB
22	EGFR	11	JAK2	7	SRD5A2	5	PSEN1	3	CSF1R	2	PRKCG
20	APP	10	HTR2A	7	FGFR1	5	STS	3	KCNH2	2	SLC5A1
20	TNF	9	SERPINE1	6	SLC6A4	5	CHEK2	3	PDGFRA	2	THRB
19	SIRT1	9	HMGCR	6	DRD2	5	BRAF	3	PTPN22	2	TNNC1
19	NR3C1	9	EDNRB	6	RET	5	FLT3	3	RPS6KA3	1	PRSS1
16	AR	9	HNF4A	6	MAOA	5	FLT4	3	FADS1	1	CA2
15	CYP3A4	9	SREBF2	6	NR1H3	4	NTRK1	3	MPL	1	TYR
										1	F10
										1	TUBB2B

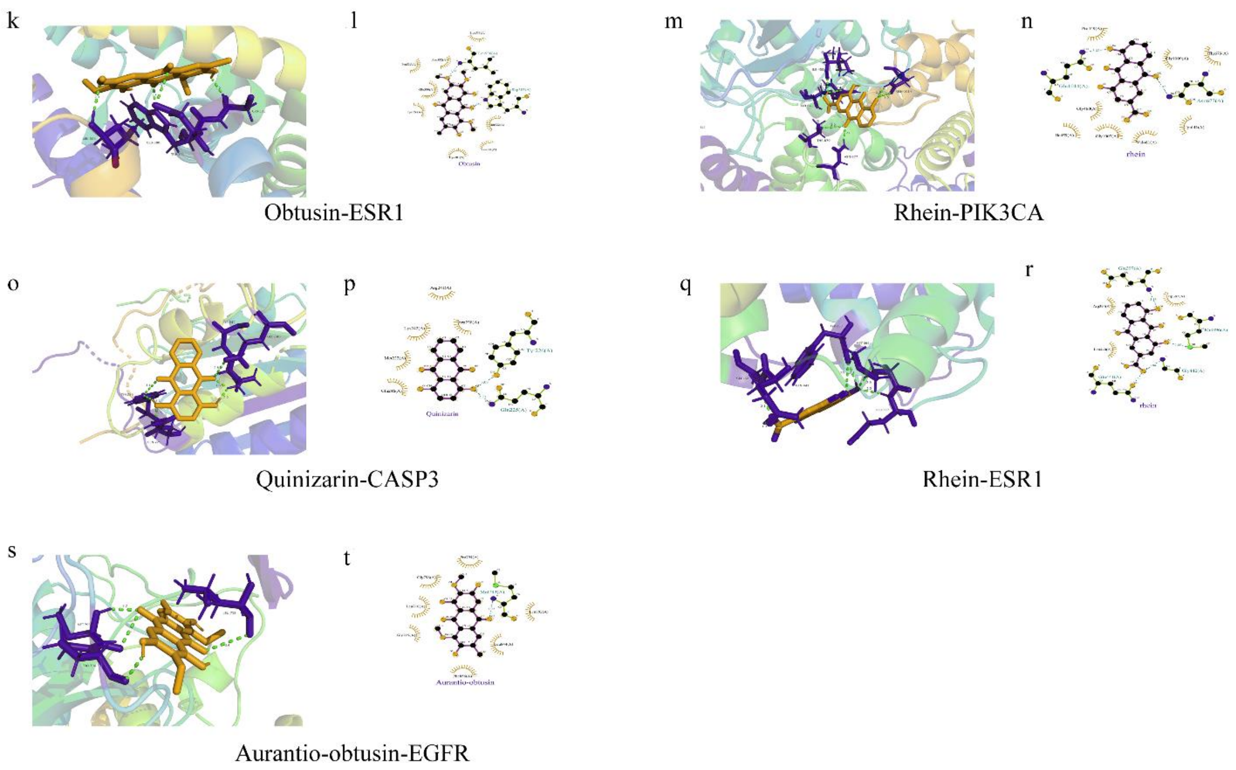

Figure 1: Molecular docking model of other drug components and core targets. Figure k, m, o, q, and s represent the molecular model of the compound is in the binding pocket of the protein. yellow represents the chemical structure of the drug component. Blue represents the part of the target that binds to the drug component. green color dotted represents the conventional hydrogen bonds between drug and target. Figure l, n, p, r, and t show the interactions between compounds and surrounding residues. In the plan, green color dotted line represents conventional hydrogen bonds.
